# Bimetallic nickel–cobalt hydrides in H_2_ activation and catalytic proton reduction†
†Electronic supplementary information (ESI) available: Experimental results, procedures and characterization including NMR spectra and crystallographic data. CCDC 1841904–1841908. For ESI and crystallographic data in CIF or other electronic format see DOI: 10.1039/c8sc04346a


**DOI:** 10.1039/c8sc04346a

**Published:** 2018-10-30

**Authors:** Xiaoxiao Chu, Jihao Jin, Bangrong Ming, Maofu Pang, Xin Yu, Chen-Ho Tung, Wenguang Wang

**Affiliations:** a Key Lab for Colloid and Interface Chemistry of Education Ministry , School of Chemistry and Chemical Engineering , Shandong University , 250100 , China . Email: wwg@sdu.edu.cn; b School of Chemistry and Materials Science , Ludong University , Yantai , 264025 , China

## Abstract

The synergism of the electronic properties of nickel and cobalt enables bimetallic NiCo complexes to process H_2_. The nickel–cobalt hydride [(dppe)Ni(pdt)(H)CoCp*]^+^ (**[1H]^+^**) arising from protonation of the reduced state **1** was found to be an efficient electrocatalyst for H_2_ evolution with Cl_2_CHCOOH, and the oxidized [Ni(ii)Co(iii)]^2+^ form is capable of activating H_2_ to produce **[1H]^+^**. The features of stereodynamics, acid–base properties, redox chemistry and reactivity of these bimetallic NiCo complexes in processing H_2_ are potentially related to the active site of [NiFe]-H_2_ases.

## Abstract 2

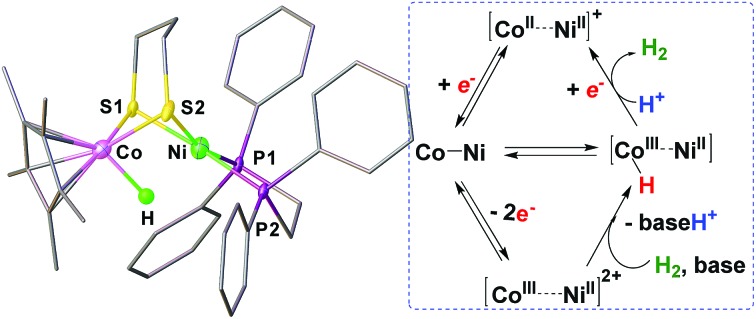
The synergism of the redox properties of nickel and cobalt enables bimetallic NiCo complexes to process H_2_.

## Introduction

**Fig. 1:**
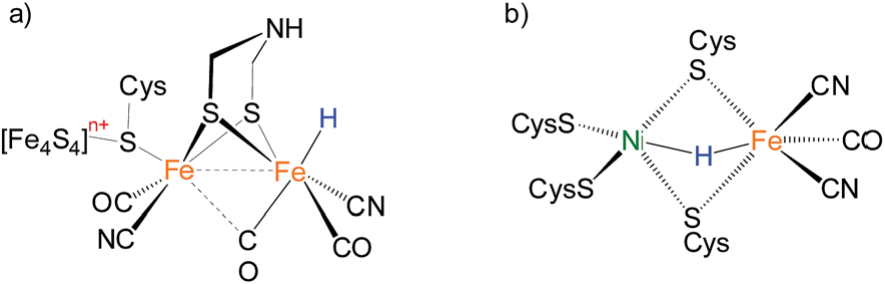
Hypothesized “hydride structure” for the active site of (a) [FeFe]-H_2_ases and (b) [NiFe]-H_2_ases.

Bimetallic complexes have emerged as important components in inorganic chemistry, homogeneous catalysis and biocatalysis.^1^–^5^ Compared to monometallic catalytic processes, bimetallic catalysis can promote the rate and selectivity of a reaction catalyzed by the first metal in the bimetallic catalyst by synergism with the electronic and steric properties of the second metal, or through a concerted activation process in which both metals participate in activation of the substrate, lowering the activation barrier.^6^,^7^ Bimetallic catalysis is common in metalloenzymes that perform multi-electron redox reactions.^8^–^10^ Examples are the active sites of [FeFe]-H_2_ases and [NiFe]-H_2_ases, which catalyze the production and uptake of dihydrogen.^11^,^12^ Both of the catalysis invoke bimetallic hydride intermediates, but the [FeFe]-H_2_ases are thought to feature terminal-hydride intermediates while [NiFe]-H_2_ases are proposed to operate through bridging hydrides in catalytically significant states (Fig. 1).^13^–^16^

**Fig. 2:**
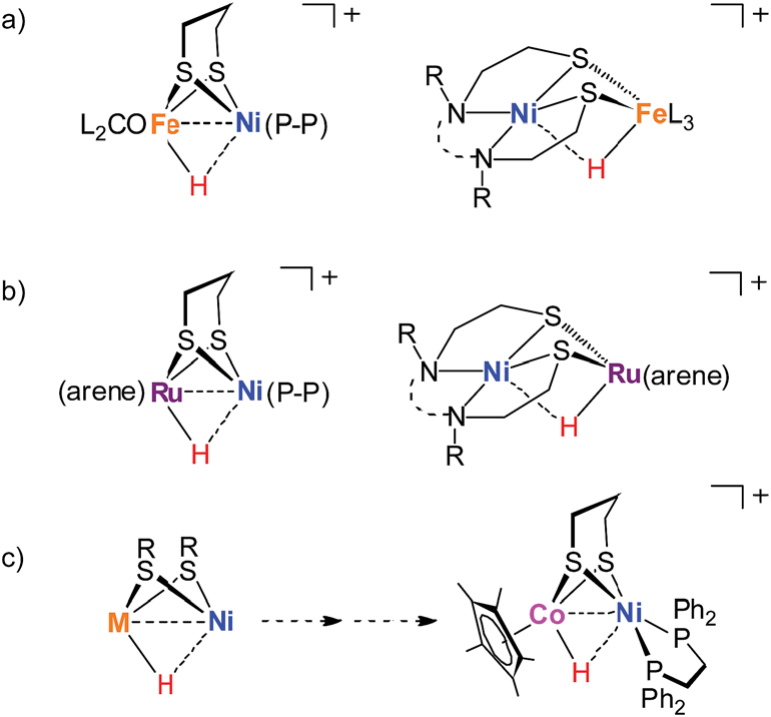
Examples of nickel-based heterobimetallic hydride complexes: (a) reported Ni(ii)–H–Fe(ii), (b) reported Ni(ii)–H–Ru(ii), and (c) Ni(ii)–H–Co(iii) described in this work.

Reflecting interest in utilization of earth abundant metals for controlling energy storage and release, functional modeling of hydrogenases continues to attract considerable attention. Compared to [FeFe]-H_2_ase mimics, modeling the active site of [NiFe]-hydrogenases has proven more challenging^17^–^23^ and very few of the models clarify the processing of H_2_.^24^–^28^ Especially, the nickel–iron hydride models [Ni(ii)HFe(ii)]^+^ remain rare although they are important in modeling research.^29^ The established [Ni(ii)HFe(ii)]^+^ models are [(dppe)Ni(pdt)(H)Fe(PR_3_)_*x*_(CO)_3–*x*_]^+^,^30^ [Ni(N_2_S_2_)Fe(H)(P(OEt)_3_)_3_]^+^,^26^ [(dppe)Ni(pdt)(H)Fe(CNBAr^F^_3_)_2_(CO)]^–^ (ref. 27) and [(pnp)Ni(pdt)(H)Fe(CO)(dppv)]^+^.^28^ They were derived from protonation of the corresponding reduced Ni(i)Fe(i) state or were generated from the activation of H_2_ with an oxidized [Ni(ii)Fe(ii)]^2+^ precursor (Fig. 2a).

Biological catalysis in a bimetallic manner offers guidance for the exploration of heteronuclear redox chemistry to precede small molecule activations under mild conditions. Four-coordinate nickel(ii) complexes such as Ni(N_2_S_2_)^24^,^31^ and (dppe)Ni(pdt)^32^,^33^ are optimal modules to assemble diverse bimetallic complexes, which expand the synthetic NiFe models to the nickel-based heterobimetallic NiM platform and encourage the exploration of fast bioinspired catalysts for the production or activation of H_2_.^31^,^34^–^37^ For example, [NiRu] complexes bearing the Ni(N_2_S_2_) metalloligand are active for H_2_ heterolysis providing [Ni(ii)HRu(ii)]^+^ hydrides with the assistance of an external base (Fig. 2b).^33^,^38^

Cobalt is earth-abundant and many cobalt complexes have been shown to be efficient catalysts for H_2_ production^11^,^39^ and hydrogenation reactions.^40^,^41^ However, bimetallic NiCo complexes have been reported only infrequently^42^ and the related heteronuclear hydride complexes have never been documented. In this work, we produced a heterobimetallic [Ni(ii)Co(iii)H]^+^ hydride [(dppe)Ni(pdt)(H)CoCp*]^+^ (**[1H]^+^**, pdt^2–^ = 1,3-(CH_2_)_3_S_2_^2–^, Cp*^–^ = Me_5_C_5_^–^, and dppe = Ph_2_PC_2_H_4_PPh_2_) by protonating the reduced state [(dppe)Ni(pdt)CoCp*] (**1**) for a catalytic proton reduction (Fig. 2c). Processing of H_2_ with a class of Ni–Co complexes, which are potentially related to the structure and properties of the [NiFe]-H_2_ase, has been studied.

## Results and discussion

### [(dppe)Ni(pdt)(Cl)CoCp*]^+^ and (dppe)Ni(pdt)CoCp*

**Scheme 1:**
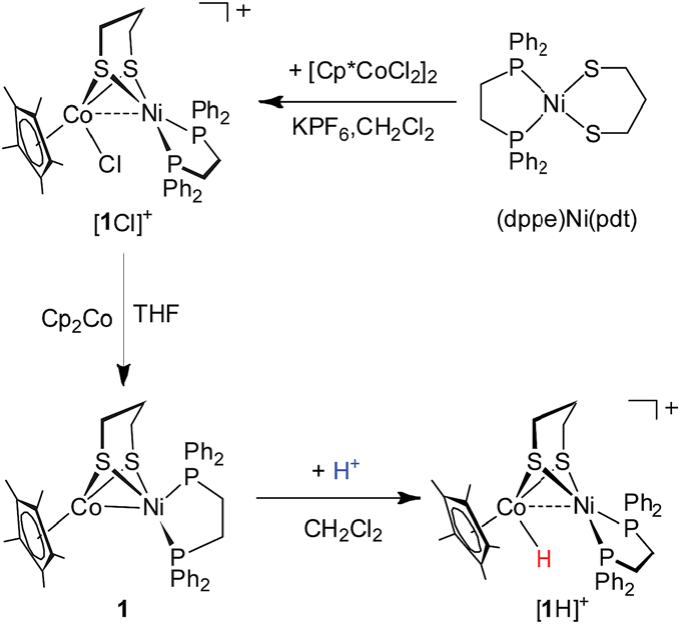
Synthesis of Ni–Co bimetallic complexes.

**Fig. 3:**
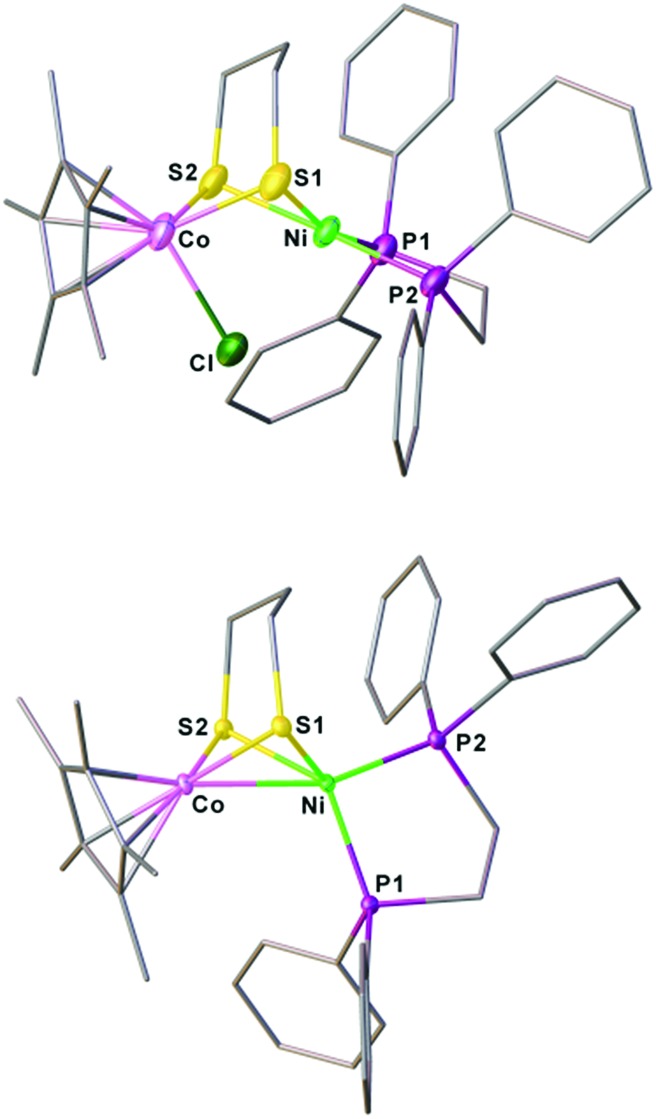
Structures of **[1Cl]^+^** and **1** with thermal ellipsoids drawn at the 50% probability level. The counter anion PF_6_^–^ in **[1Cl]^+^** and all hydrogen atoms are omitted for clarity. Selected distances (Å): for **[1Cl]^+^**, Co–Ni, 2.926(2); Co–Cl, 2.316(2); Co–S1, 2.252(2); Co–S2, 2.259(2); Ni–S1, 2.258(2); Ni–S2; 2.247(2). For **1**, Co–Ni, 2.4722(5); Co–S1, 2.1947(7); Co–S2, 2.1820(7); Ni–S1, 2.2645(7); Ni–S2; 2.2268(7).

The synthesis of a heteronuclear Ni(ii)Co(iii) dithiolate **[1Cl]^+^** entailed the assembly of (dppe)Ni(pdt) to [Cp*CoCl_2_]_2_. Treatment of [Cp*CoCl_2_]_2_ with two equiv. of (dppe)Ni(pdt) and KPF_6_ solids in CH_2_Cl_2_ results in the formation of the product **[1Cl]^+^** in a high yield (Scheme 1). The ^31^P NMR spectrum of **[1Cl]^+^** has a sharp singlet at *δ* 54.4 corresponding to dppe, indicating that the two phosphine groups are chemically equivalent. Crystallographic analysis of **[1Cl]^+^** reveals that the Ni center adopts a square-planar geometry (Fig. 3) and is linked to the Cp*Co fragment through the pdt^2–^ ligand. The Ni···Co distance of 2.926(2) Å far exceeds the sum of the covalent atomic radii of Ni (1.24 Å) and Co (1.26 Å, low-spin).^43^

Reduction of the cationic Ni(ii)Co(iii) complex **[1Cl]^+^** with two equivalents of Cp_2_Co affords the reduced compound **1**. It dissolves well in nonpolar solvents such as toluene or benzene to form deep red solutions, which is very air sensitive and decomposes into insoluble species. Crystallographic analysis of **1** confirms a neutral complex with the formula (dppe)Ni(pdt)CoCp* (Fig. 3). The structure of **1** is more compact than that of **[1Cl]^+^** and this is reflected by the Ni–Co distance, which is 0.454 Å shorter after the 2e^–^ reduction. The Ni–Co distance of 2.4722(5) Å in **1** is comparable to the Ni–Fe distance of 2.4666(6) Å in (dppe)Ni(pdt)Fe(CO)_3_.^17^ Given the sum of the covalent atomic radii of Ni and Co, the Ni–Co separation suggests a metal–metal bond. The 2e^–^ reduction causes the Cp*–Co distance to decrease from 1.699 Å to 1.686 Å. Since the Ni center adopts an approximately tetrahedral geometry, compound **1** can be appropriately considered as a Ni(i)Co(ii) species in the solid state.

**Fig. 4:**
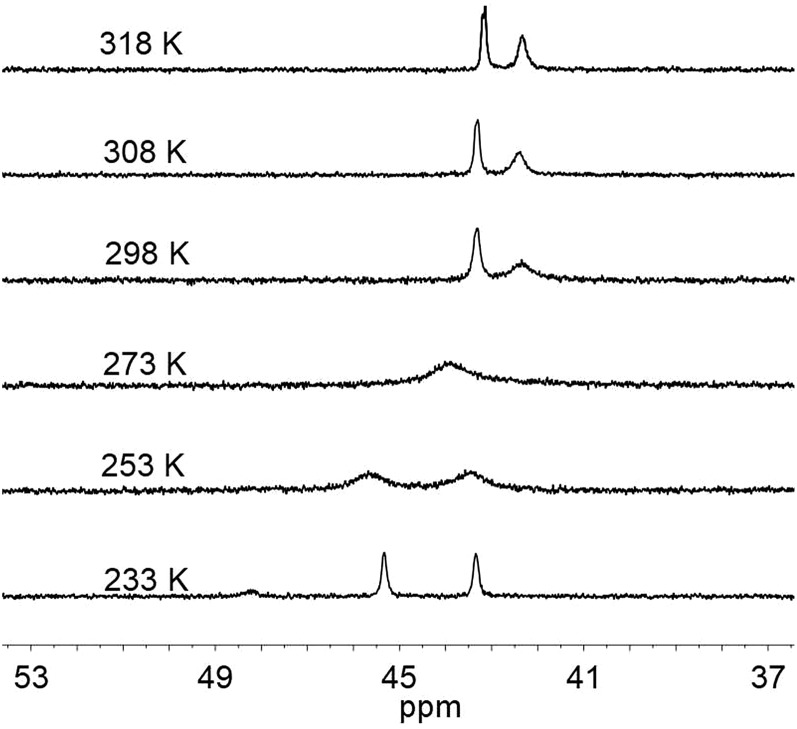
^31^P NMR spectra of **1** recorded at various temperatures in toluene.

Interestingly, the ^31^P NMR spectroscopic analysis shows that compound **1** exhibits two isomers in solution at room temperature (eqn (1)). The phosphorus resonance signals appeared as two broad signals at *δ* 42.9 and 41.8 in a ratio of approximately 1 : 1 (Fig. 4). When the temperature was increased to 318 K, the broad resonance at *δ* 41.8 became resolved but the ratio of the two signals was maintained. Decreasing the temperature to 253 K, the two resonances coalesced into a single broad peak at *δ* 44. At this lower temperature, however, the broad signal decoalesced into two peaks at 45.7 and 43.6 ppm, which is thought to be consistent with the tetrahedral geometry of Ni that was observed in the solid state structure. Overall, the results of ^31^P NMR observations at temperatures ranging from 233 K to 298 K reveal a dynamic process in which the ^31^P sites in dppe are interchanged. The rotation of dppe at the Ni site could proceed through an intermediate or transition state with square-planar Ni.30*b* It is more likely that in solution at room temperature, **1** consists of two isomers with resonance states of Ni(i)Co(ii) and Ni(ii)Co(i). Such rotation-induced redox behavior has also been described by Rauchfuss *et al.*30*a* for (CO)_3_Fe(pdt)Ni(dppe). In addition, the reduced NiRu compound (cymene)Ru(pdt)Ni(dppe) features a rigid tetrahedral Ni(0) center.33*a*1
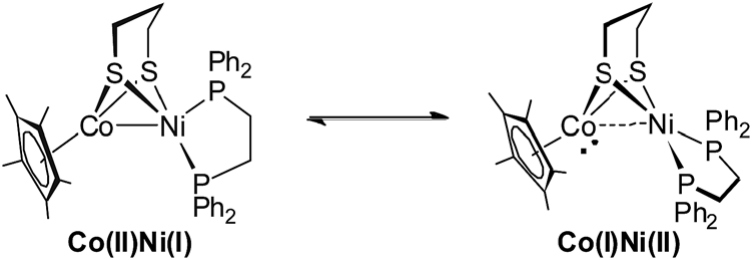



### Protonation of (dppe)Ni(pdt)CoCp*

The reduced compound **1** undergoes protonation, affording the [Ni(ii)Co(iii)H]^+^ hydride complex **[1H]^+^**. Addition of an equivalent amount of an acid such as [HPPh_3_]BF_4_ (p*K*MeCNa = 8.0)^44^ or Cl_2_CHCOOH (p*K*MeCNa = 13.2)^45^ to solutions of **1** in CH_2_Cl_2_ immediately leads to the color changing from brown to dark. The resulting complex **[1H]^+^** was isolated by filtration after dilution of the reaction solution with Et_2_O. The ^1^H NMR spectrum of **[1H]^+^** features a hydride signal at *δ* –9.77 as a singlet (Fig. S9†). No ^31^P coupling was resolved, indicating that the hydride is located at the Co(iii) center rather than being bound to Ni.^46^ The ^31^P signal of the dppe group appears as a singlet at *δ* 67.5, about 13.1 ppm down-field shifted relative to that of **[1Cl]^+^** (Fig. S8†). An alternative approach to synthesize **[1H]^+^** is by displacement of the Cl^–^ ligand of **[1Cl]^+^** by a hydride ligand donated from NaBH_4_.

**Fig. 5:**
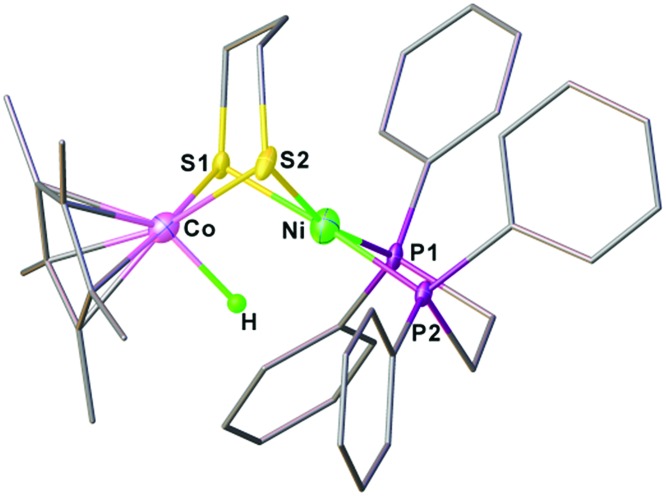
Structure of **[1H]^+^**. Selected bond distances (Å): Co–Ni, 2.556(1); Co–H, 1.45(5); Co–S1, 2.22(1); Co–S2, 2.225(1); Ni–S1, 2.222(1); Ni–S2, 2.247(1).

Crystallographic analysis agrees with the NMR spectroscopic assignments of the nickel–cobalt dithiolates as a hydride. The framework of [(dppe)Ni(pdt)(X)CoCp*]^+^ is very similar to that of **[1Cl]^+^** (Fig. 5). The striking difference is the Ni···Co separation (2.556(1) Å), which is 0.37 Å shorter than that in **[1Cl]^+^**. Protonation causes the Ni–Co distance to increase only by 0.1 Å, which is consistent with the metal–metal bond changes associated with the protonation of reduced NiFe models.30*a* The hydride ligand was located and refined and the Co–H bond length of 1.45(5) Å is much shorter than the Ni–H bond length (1.91(5) Å), suggesting that the hydride is strongly coordinated at the Co center instead of adopting a bridging position between the bimetallic centers.

Given the rotation-induced redox behavior of the reduced compound, we propose that the protonation reaction proceeds *via* the mixed-valent conformer of Ni(ii)Co(i). With dppe rotation, nickel is stabilized in the oxidized Ni(ii) form with concomitant cleavage of the metal–metal bond resulting in cobalt being in a strongly reduced Co(i) state.^29^,30b The formation of the [Ni(ii)Co(iii)H]^+^ hydride arises from protonation of the Cp*Co site rather than the metal–metal bond. This “redox isomerization–protonation” mechanism is close to that proposed for (CO)_3_Fe(pdt)Ni(dppe) and reflects the redox-flexibility of the (dppe)Ni(pdt) module.^30^

### Acidity of **[1H]^+^**

The acidic character of the hydride ligand in **[1H]^+^** was manifested by the H/D exchange reaction with D_2_O giving **[1D]^+^** solely and with no liberation of HD or H_2_. After addition of excess D_2_O (20 equiv.) to the CD_3_CN solution (0.6 mL) of **[1H]^+^** in a J. Young tube, the reaction process was monitored by NMR spectroscopy. ^1^H NMR spectroscopic analysis showed that the hydride signal disappears slowly, while the ^31^P resonance remains as a singlet at *δ* 67.4. The appearance of a Co–D signal was observed at *δ* –9.66 in the ^2^H NMR spectrum (Fig. S14†). In agreement with the calculated isotopic distribution of **[1D]^+^**, ESI-MS spectral analysis featured a characteristic peak at *m*/*z* = 758.1216 for **[1D]^+^***vs. m*/*z* = 757.1175 for **[1H]^+^**. The exchange reaction between **[1H]^+^** and D_2_O is relatively slow. A plot of the integrated hydride signal *vs.* time suggests that the reaction follows unimolecular kinetics and has a rate constant of 5.023 × 10^–5^ s^–1^ at 25 °C (*t*_1/2_ = 3.83 h, Fig. S16†).2
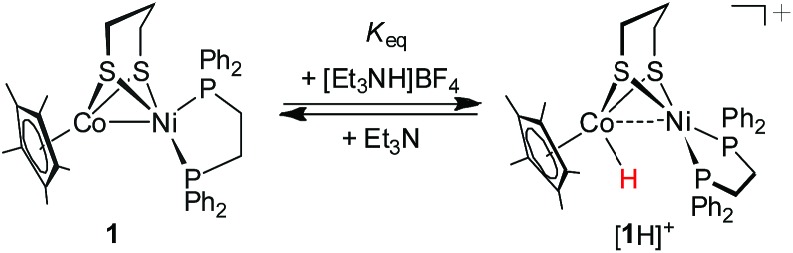



This bimetallic [Ni(ii)Co(iii)H]^+^ hydride was found to be much less acidic than the reported [Ni(ii)(H)Fe(ii)]^+^ hydrides such as (CO)_3_Fe(pdt)Ni(dppe) (p*K*MeCNa = 10.7) and (CO)_2_(PPh_3_)Fe(pdt)Ni(dppe) (p*K*MeCNa = 14.9),30*a* and the diiron bridging hydride [(μ-H)Fe(pdt)(PMe_3_)_2_(CO)_4_]^+^ (p*K*MeCNa = 12).^47^ The acidity of **[1H]^+^** was further evaluated by the protonation of **1** with [Et_3_NH]BF_4_ (p*K*MeCNa = 18.8).^44^ An equilibrium was established between **1** and 1.0 equiv. of [Et_3_NH]BF_4_ in PhCN (eqn (2)), which provided an equilibrium mixture of **[1H]^+^** and **1** in a ratio of 0.22 : 1 as determined from the ^31^P NMR spectrum. A p*K*_a_ of 17.5 was estimated for **[1H]^+^** on the basis of the calculated equilibrium constant *K*_eq_ (0.05) (Fig. S17†). The results indicate that the acidity of **[1H]^+^** is weak, while its conjugated base, the Ni(ii)Co(i) complex, exhibits a strong basic character.

### Homolytic and heterolytic bond energies for NiCo–H

**Scheme 2:**
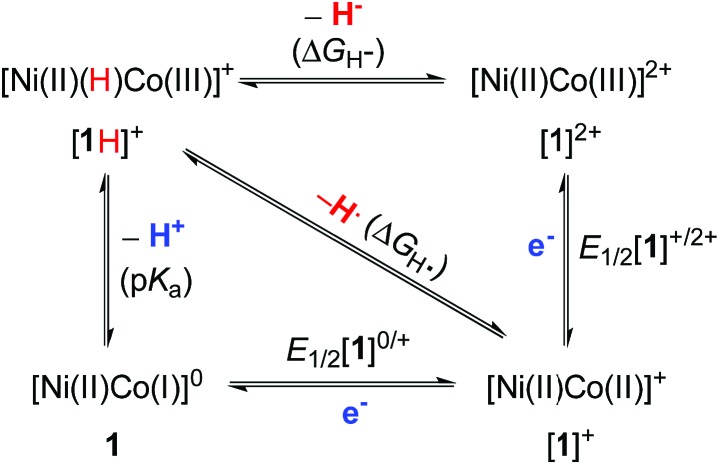
Schematic pathways to the cleavage of the NiCo–H bond in **[1H]^+^**.

Because of the centrality of metal hydrides in catalytic H_2_ production, we determined the homolytic and heterolytic bond energies for NiCo–H in an effort to learn more about the thermodynamic properties of the [Ni(ii)(H)Co(iii)]^+^ hydride. The cleavage of the M–H bond can involve transfer of electrons, a proton, a hydrogen atom or a hydride^48^–^51^ and is very dependent on the bond energy. Scheme 2 illuminates the relationship between acidity (p*K*_a_), homolytic bond dissociation energy (BDFE, Δ*G*_H˙_), thermodynamic hydricity (Δ*G*_H^–^_), and redox potentials (*E*_1/2_) for the NiCo–H cleavage.

3 Δ*G*_H˙_ = 1.37 p*K*_a_ + 23.06 *E*_1/2_**[1]^0/+^** + 54.9

4 Δ*G*_H^–^_ = 1.37 p*K*_a_ + 23.06 *E*_1/2_**[1]^0/+^** + 23.06 *E*_1/2_**[1]^+/2+^** + 79.6

According to the thermodynamic cycle, the redox potentials of the **[1]^0/+^** and **[1]^+/2+^** couples are required to calculate Δ*G*_H˙_ and Δ*G*_H^–^_. Cyclic voltammetry on PhCN solutions of **1** indicates oxidations at *E*_1/2_ = –0.45 V (*i*_pa_/*i*_pc_ = 1) and *E*_1/2_ = –0.01 V (quasi-reversible, Fig. S19†). These couples are assigned as one-electron processes for **[1]^0/+^** and **[1]^+/2+^**, respectively. The direct evidence is derived from oxidations conducted on a preparative scale (see below). Using the thermodynamic equations derived from eqn (3) and (4),30a,^48^,^49^ Δ*G*_H˙_ and Δ*G*_H^–^_ are calculated to be 69 and 93 kcal mol^–1^, respectively.

### Oxidizing **1** for H_2_ activation

Oxidation of **1** with one equiv. of FcBF_4_ provided a cationic complex **[1]^+^**, while using AgBF_4_ (*E*_1/2_ = 0.65 V, CH_2_Cl_2_)^52^ allowed for the further oxidization of **[1]^+^** to **[1]^2+^**. The formation of a new diamagnetic species was signaled by a ^31^P peak at *δ* 59.5 (s) (Fig. S20†). As a 32e^–^ bimetallic species, **[1]^2+^** is unstable and cannot be isolated. When the reaction was conducted in the presence of MeCN, the dicationic complex [Ni(ii)Co(iii)]^2+^ was stabilized in the form of [(dppe)Ni(pdt)(MeCN)CoCp*]^2+^ (**[1(NCMe)]^2+^**).

**Fig. 6:**
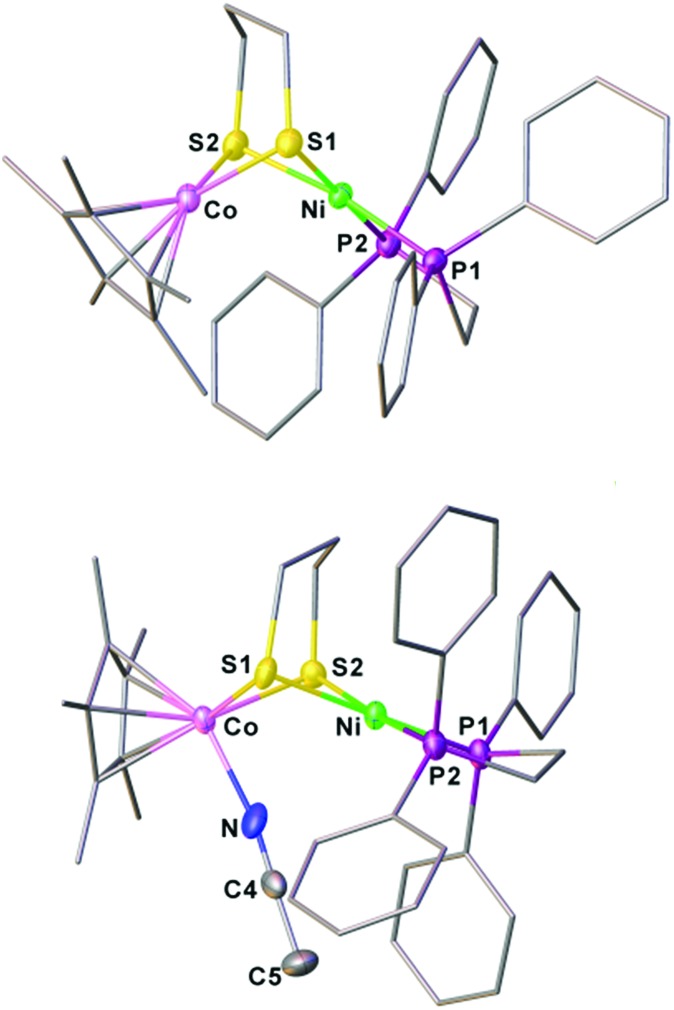
Structures of **[1]^+^** and **[1(NCMe)]^2+^** with thermal ellipsoids drawn at the 50% probability level. BF_4_^–^ anions, hydrogen atoms and solvent are omitted for clarity. Selected bond distances (Å): for **[1]^+^**, Co–Ni, 2.657 (1); Co–S1, 2.210(1); Co–S2, 2.215(1); Ni–S1, 2.239(1); Ni–S2, 2.229(1); for **[1(NCMe)]^2+^**, Co–Ni, 3.005; Co–S1, 2.246(1); Co–S2, 2.263(1); Ni–S1, 2.237(1); Ni–S2, 2.239(1).

The structures of **[1]^+^** and **[1(NCMe)]^2+^** were characterized crystallographically. In both structures, the Ni center adopts a square-planar coordination geometry (Fig. 6). In **[1(NCMe)]^2+^**, the MeCN is bound to the Co, consistent with electrochemical assignment that the second oxidation occurs at the Cp*Co site. The 1e^–^ oxidation increases the Ni···Co distance from 2.6572(10) Å for **[1]^+^** to 3.005 Å for **[1(NCMe)]^2+^**. The principal change in the oxidation of **1** is the coordination sphere of nickel, which turns from roughly tetrahedral to a strictly square-planar geometry. According to the structures of **1** and **[1]^+^**, the first oxidation event occurs at the Ni(dppe) site. Related to **1**, the Ni···Co distance in **[1]^+^** is longer by 0.18 Å, meanwhile the Cp*–Co distance increases slightly from 1.686 Å to 1.693 Å.

**Scheme 3:**
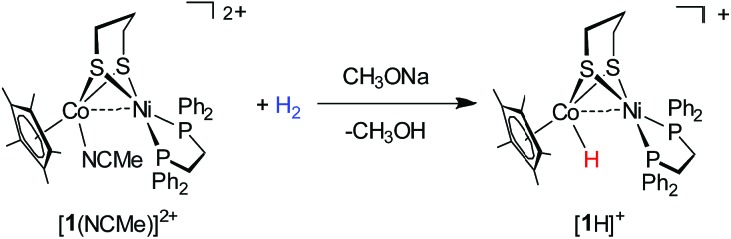
Hydrogen activation by **[1(NCMe)]^2+^**.

The high affinity of the dicationic [Ni(ii)Co(iii)]^2+^ complex toward the hydride was further demonstrated by the reaction of activating H_2_ (Scheme 3). The MeCN solution of **[1(NCMe)]^2+^** contained in a Schlenk flask was bubbled with H_2_ (10 psi) for 3 min, and then a solution of CH_3_ONa in MeOH was added (Fig. S24†). Over the course of 10 min at room temperature, the reaction mixture turns from red to dark brown. As confirmed by ^1^H NMR and ^31^P NMR spectra, the organometallic product is **[1H]^+^**. Interestingly, in addition to CH_3_ONa, alkali metal carbonates such as Na_2_CO_3_ can be employed as the base to assist **[1(NCMe)]^2+^** to activate H_2_.

### Electrocatalytic H_2_ production

**Fig. 7:**
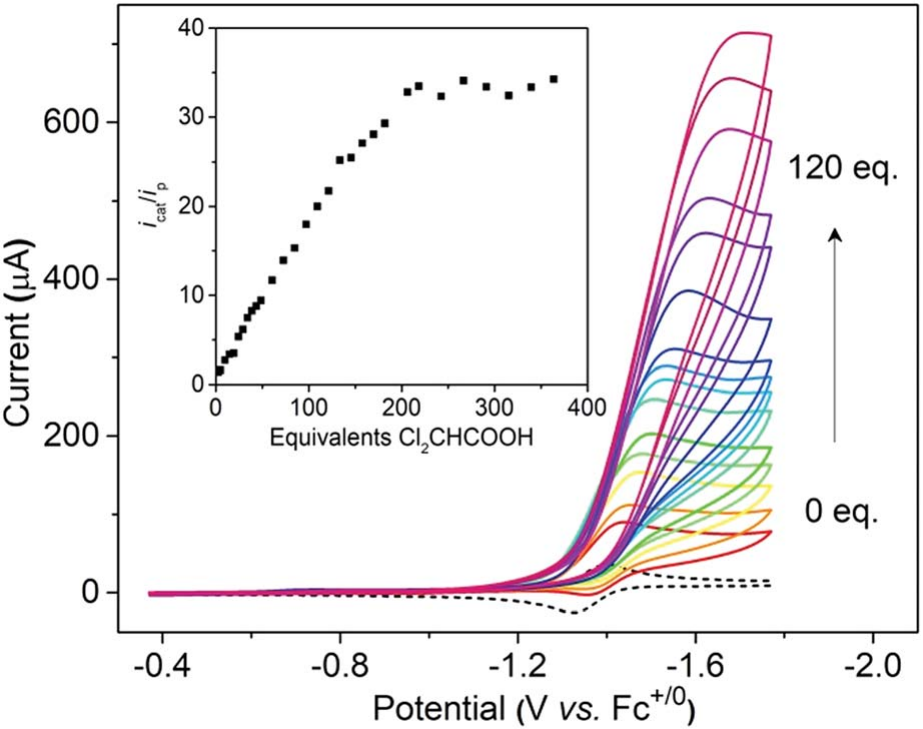
Cyclic voltammograms of **[1H]^+^** with increments of Cl_2_CHCOOH (from 0 to 120 equiv.), inset: plot of catalytic current *i*_c_/*i*_p_ for **[1H]^+^***vs.* equivalent of Cl_2_CHCOOH. Conditions: 1 mM **[1H]^+^** in MeCN, 0.1 M *n*-NBu_4_PF_6_, scan rate = 100 mV s^–1^, and V *vs.* Fc^0/+^.

Cyclic voltammetry indicates that **[1H]^+^** only undergoes a reversible reduction process at –1.36 V (*i*_pc_/*i*_pa_ = 0.98), which is tentatively assigned to the [Ni(ii)Co(iii)H]^+^/[Ni(ii)Co(ii)H]^0^ couple (Fig. S26†). To evaluate the catalytic activity of **[1H]^+^** as an electrocatalyst for H_2_ production, Cl_2_CHCOOH (*E*^0^ = –0.92 V)^45^ was selected as the proton source. Upon the addition of Cl_2_CHCOOH to the CH_2_Cl_2_ solution of **[1H]^+^**, the reduction events for **[1H]^+/0^** became irreversible, and the cathodic current intensity increased linearly with sequential increasing of the acid concentration (Fig. 7, inset). These observations are consistent with the aspects of proton reduction catalysis.^53^,^54^ Plots of *i*_c_/*i*_p_*vs.* [Cl_2_CHCOOH] are linear up to 218 equiv. of acid, indicating that the catalysis is second order with respect to the acid. The turnover frequency was estimated to be 244 s^–1^ (Table S1†). In the controlled experiment, proton reduction with a glassy carbon electrode was performed at a potential nearly 0.37 V more negative than that in the catalysis performed with **[1H]^+^** (Fig. S25†).

**Scheme 4:**
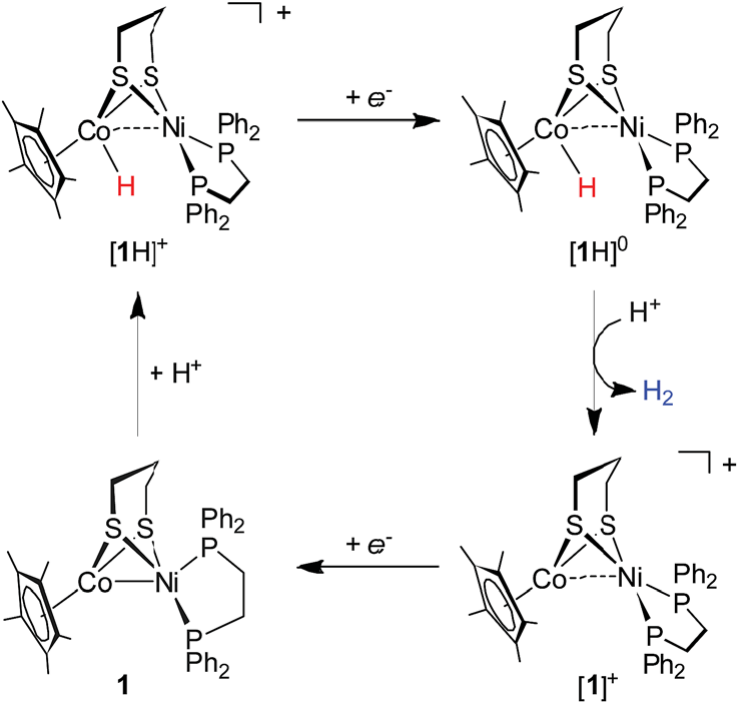
Proposed catalytic cycle for proton reduction by **[1H]^+^**.

To gain insight into the catalytic mechanism, we examined the chemical reduction of **[1H]^+^** with Cp*_2_Co (*E*_1/2_ = –1.94 V in CH_2_Cl_2_). The reaction was indicated by an immediate change of the color of the solution from brown to black. Efforts to isolate and characterize the reduced **[1H]^0^** were unsuccessful since it is converted to **1** and loses H_2_ in a matter of minutes. Addition of protic acids such as Cl_2_CHCOOH or H(OEt_2_)_2_BAr^F^_4_ into a solution of **[1H]^0^** in THF, generated *in situ* by reaction of **[1H]^+^** with Cp*_2_Co, resulted in the formation of **[1]^+^** and release of H_2_. The yield of H_2_, quantified by GC analysis, is 91% ± 5 (in three experiments) and is close to the stoichiometric value. We propose that the catalytic H_2_ evolution is based on the reduction of the [Ni(ii)Co(iii)H]^+^ hydride, operating through an ECEC mechanism (Scheme 4).

## Conclusions

A class of new NiCo complexes, relevant to the active site models of [NiFe]-H_2_ases, have been studied and their features of stereodynamics, acid–base properties, redox chemistry and reactivity are disclosed. Incorporating the (dppe)Ni(pdt) module with a Cp*Co fragment enables the bimetallic NiCo complexes to achieve Co-centered H_2_ evolution and uptake. Such a bimetallic way can disperse the strong effects of redox over the two metal sites.^29^ Owing to the redox flexibility of Ni(dppe), distortion at the Ni center of the reduced state affects the oxidation state of the bimetallic centers, notably interconversion of Ni(i)Co(ii) and Ni(ii)Co(i). The protonation reaction can proceed *via* the mixed valence isomer Ni(ii)Co(i) with enhanced basicity to afford [Ni(ii)Co(iii)H]^+^. The bimetallic hydride is catalytically active and was demonstrated to be an efficient electrocatalyst for the reduction of weak protic acid at a mild potential. Despite a stoichiometric reaction, the oxidized state [Ni(ii)Co(iii)]^2+^ is capable of activating H_2_ to give the hydride.

Bimetallic hydride species are commonly encountered in hydrogenase modeling. Bimetallic active sites in hydrogenases provide an elegant means of softening the effect of redox reactions on the acid–base properties of the hydride.30*a* In addition to biocatalysis, metal hydrides play a central role in a variety of chemical transformations.^55^–^59^ Studies of metal hydride chemistry are essential to control the elementary steps for catalysis.^60^–^62^ However, the related thermodynamic properties of bimetallic systems have been inadequately examined to date.

## Conflicts of interest

There are no conflicts to declare.

## Supplementary Material

Supplementary information

Crystal structure data
