# Supplementary material for: Bimetallic nickel–cobalt hydrides in H2 activation and catalytic proton reduction
Source: Chem Sci. 2018 Oct 30;10(3):761–7. doi: 10.1039/c8sc04346a (PMC6340403; doi:10.1039/c8sc04346a)
Supplement: Supplementary file 1 [file SC-010-C8SC04346A-s001.pdf]

Electronic Supplementary Material (ESI) for Chemical Science.
This journal is © The Royal Society of Chemistry 2018

Bimetallic Nickel–Cobalt Hydride in H_2 Activation and Catalytic Proton Reduction

Xiaoxiao Chu,[a,b] *Jihao Jin,*[a] *Bangrong Ming,*[a] *Maofu Pang,*[a] *Xin Yu,*[a] *Chen-Ho Tung*[a] *and Wenguang Wang**[a]

[a]School of Chemistry and Chemical Engineering, Shandong University, Jinan, 250100, China

[b]School of Chemistry and Materials Science, Ludong University, Yantai, 264025, China.

Table of contents

1. **General information** 3

2. **Experimental procedures** 4

3. **Figures** 7

Fig. S1-3 NMR spectra and ESI-MS spectrum of [**1**Cl]$^+$ 7

Fig. S4-7 NMR spectra and ESI-MS spectrum of **1** 10

Fig. S8-10 NMR spectra and ESI-MS spectrum of [**1**H]$^+$ 14

Fig. S11-12 VT-NMR spectra of [**1**H]$^+$ 17

Fig. S13-15 NMR spectra and ESI-MS spectrum of [**1**D]$^+$ 19

Fig. S16 Plot of the hydride integration vs time for the reaction of [**1**H]$^+$ with excess D_2O 22

Fig. S17 ^{31}P NMR spectrum of **1** reacts with 1 equiv of [Et_3NH]BF_4 23

Fig. S18 ESI-MS spectrum of [**1**]$^+$ 24

Fig. S19 Cyclic voltammogram of **1** recorded in PhCN 25

Fig. S20 ^{31}P NMR spectrum of [**1**]$^{2+}$ 26

Fig. S21-22 NMR spectra and ESI-MS spectrum of [**1**(NCMe)]$^{2+}$ 27

Fig. S23 ESI-MS of [(dppe)Ni(pdt)Co(NCMe)Cp*]$^{2+}$, [**1**(NCMe)]$^{2+}$ 29

Fig. S24 Control experiments of H_2 splitting 30

Fig. S25 Cyclic voltammograms of $Cl_2CHCOOH$ solution after the addition of [**1**H]$^+$ 31

Table S1. Selected parameters of electrochemical H_2 production.. 32

Fig. S26 Cyclic voltammogram for the couple [**1**H]$^{+/0}$.. 33

Fig. S27 Cyclic voltammogram of [**1**H]$^{+}$ at various scan rate for the couples [**1**H]$^{+/0}$.................... 34

Fig. S28 ^{1}H NMR spectrum of [**1**H]$^{+}$ with excess $Cl_2CHCOOH$.. 35

4. X-ray crystal structure analysis 36

1. General information

All reactions were carried out under dry nitrogen atmosphere using standard Schlenk techniques. All reagents were purchased from Sigma–Aldrich, and used as received. All solvents were stored under nitrogen. All NMR spectra were recorded in J. Young NMR tubes using Bruker Avance 500 spectrometers. $^{31}P\{^{1}H\}$ NMR spectra were referenced to external 8% H_3PO_4 as internal standards. Cyclic voltammetry was measured by a CHI 760e electrochemical workstation (Shanghai Chen Hua Instrument Co., Ltd.) under nitrogen at room temperature (electrode types: a glassy carbon electrode as working electrode, Pt wire electrode as counter electrode, and Ag wire electrode as reference electrode). (dppe)Ni(pdt) and $[Cp^{*}CoCl_2]_2$was prepared according to the reported methods.[1,2]

2. Experimental procedures

[(dppe)Ni(pdt)(Cl)CoCp*]$^+$, [1Cl]$^+$. Ni(pdt)(dppe) was added to a dark green solution of $[Cp^*CoCl_2]_2$ (197 mg, 0.42 mmol) in 60 mL of CH_2Cl_2 (473 mg, 0.84 mmol), followed by the addition of KPF_6 (200 mg, 1.08 mmol). After stirring for 5 h, the brown solution was collected by filtration and concentrated to 5 mL. Then 30 mL of hexane was added the concentrate to afford brown precipitate. The precipitate was washed with 3×10 mL of hexane, and dried under vacuum. Yield: 650 mg (83 %). ^{1}H NMR (CD_3CN): δ 7.51−7.86 (m, 20H, C_6H_5), 2.45 (m, 2H, PCH_2), 2.31 (m, 3H, SCH_2CH_2), 2.05 (m, 2H, PCH_2), 1.88 (m, 1H, SCH_2CH_2), 1.45 (m, 2H, SCH_2). 1.31 (s, 15H, C_5H_{15}). ^{31}P{^{1}H} NMR (CD_3CN): δ 54.4 (s). ESI-MS: calcd for [**1**Cl]$^+$, 791.0812; found, 791.0796. Anal. Calcd. for $C_{39}H_{45}P_3S_2F_6CoNiCl$: C, 49.94; H, 4.84. Found: C, 50.62; H, 4.97.

[(dppe)Ni(pdt)CoCp*], 1. A mixture of [**1**Cl]$^+$ (243 mg, 0.26 mmol) and Cp_2Co (98 mg, 0.52 mmol) was stirred in 35 mL of THF at room temperature for 15 min to obtain red brown solution. And the solvent was removed under reduced pressure. The residue was extracted by ~ 5 mL of toluene through a pad of Celite and the extract was layered with 50 mL of hexane to yield black crystals at −30 °C. Yield: 120 mg (61 %). ^{1}H NMR (CD_2Cl_2): δ 7.33−7.88 (m, 20H, C_6H_5), 2.07 (m, 2H, PCH_2), 1.90 (m, 4H, SCH_2CH_2), 1.76 (m, 2H, PCH_2), 1.45 (s, 15H, C_5H_{15}) 1.31 (m, 2H, S SCH_2CH_2). ^{31}P{^{1}H} NMR (CD_2Cl_2): δ 42.7 and 42.2 (br, dppe). ESI-MS: calcd for **1**, 756.1123; found, 756.1090. Anal. Calcd. for $C_{39}H_{45}P_2S_2CoNi$: C, 61.84; H, 5.99. Found: C, 62.51; H, 6.23.

[(dppe)Ni(pdt)(H)CoCp*]$^+$, [1H]$^+$. Method A, Complex **1** (151 mg, 0.20 mmol) was dissolved into 20 mL of THF and $Cl_2CHCOOH$ (16.5 μL, 0.20 mmol) was added to give dark brown solution. After stirring 15 min, the solution was filtered through celite and dried under reduced pressure. And the product was washed with 3×10 mL of hexane. Single crystal was obtained by layering hexane into CH_2Cl_2 solution at −30 °C. Yield: 150 mg (89 %). **Method B,** To a solution of [**1**Cl]$^+$ (295 mg, 0.32 mmol) in 30 mL of acetonitrile was added a solution of $NaBH_4$ (12 mg, 0.32 mmol) in 10 mL of ethanol, immediately producing a dark brown solution. The solution was filtered through Celite, and concentrated under reduced pressure. 50 mL of Et_2O was added to obtain the precipitate. The solid was dried under vacuum. Yield: 220 mg (77 %). ^{1}H NMR (CD_2Cl_2): δ 7.55−7.76 (m, 20H, C_6H_5), 2.54

(m, 2H, PCH_2), 2.50 (m, 2H, PCH_2), 2.46 (m, 2H, SCH_2CH_2), 1.83 (m, 2H, SCH_2CH_2), 1.73 (m, 2H, SCH_2CH_2), 1.49 (s, 15H, C_5H_{15}), –9.76 (1H, br, Co(*H*)Ni). $^{31}P\{^1H\}$ NMR (CD_2Cl_2): δ 67.5 (s). ESI-MS: calcd for [**1**H]$^+$, 757.1202; found, 757.1175. Anal. Calcd. for $C_{39}H_{46}P_3S_2F_6CoNi$: C, 51.85; H, 5.13. Found: C, 52.24; H, 5.30.

Reaction of [1H]$^+$ with D_2O. A solution of [**1**H]$^+$ (20 mg, 0.022 mmol) in 0.5 mL of d_3-acetonitrile was prepared in a J. Young NMR tube. The sample was stored at room temperature and 10 equiv. of D_2O was added. The ^1H NMR spectrum was recorded each 30 min and the hydride signal (δ –9.65) diminished gradually, indicating the formation of deuterated species. The $^{31}P\{^1H\}$ NMR spectrum had no obvious change. ^2H NMR (CH_2Cl_2): δ –9.66 (1D, br, Co(*D*)Ni), $^{31}P\{^1H\}$ NMR (D_2O): 67.38 (s). ESI-MS: calcd for [**1**D]$^+$, 758.1264; found, 758.1216.

[(dppe)Ni(pdt)CoCp*]$^+$, [1]$^+$. 1 (50 mg, 0.066 mmol) was dissolved into 10 mL of acetonitrile and the solution of $AgBF_4$ (13 mg, 0.066 mmol) in 3 mL of acetonitrile was dropwise added. After 5 min, the solution was the solution was filtered through Celite and concentrated into 2 mL. The product was precipitated by the addition of 15 mL of Et_2O. Yield: 45 mg (81 %). ESI-MS: calcd for [**1**]$^+$, 756.1123; found, 756.1088. Anal. Calcd. for $C_{39}H_{45}P_2S_2BF_4CoNi$: C, 55.48; H, 5.37. Found: C, 55.90; H, 5.94.

[(dppe)Ni(pdt)CoCp*]$^{2+}$, [1]$^{2+}$. To a solution of **1** (50 mg, 0.066 mmol) in 10 mL of CH_2Cl_2 was added $AgBF_4$ (26 mg, 0.133 mmol). After 15 min, the solvent was removed under reduced pressure and the product was washed with 3×10 mL of hexane. Yield: 53 mg (86 %). $^{31}P\{^1H\}$ NMR (D_2O): 59.5 (s).

[(dppe)Ni(pdt)Co(NCMe)Cp*]$^{2+}$, [1(NCMe)]$^{2+}$. Complex [**1**]$^{2+}$ (25 mg, 0.054 mmol) was dissolved into 5 mL of acetonitrile, 20 mL of Et_2O was added to form a needle-like crystal. Yield: 20 mg (77 %). ^1H NMR (CD_2Cl_2): δ 7.65–7.82 (m, 20H, C_6H_5), 3.11 (m, 2H, PCH_2), 2.77 (m, 2H, PCH_2), 2.66 (m, 1H, SCH_2CH_2), 2.58 (m, 3H, SCH_2CH_2), 2.35 (m, 2H, SCH_2), 2.17 (s, 3H, CH_3CN), 1.99 (s, 15H, C_5H_{15}) $^{31}P\{^1H\}$ NMR (CD_2Cl_2): δ 54.69 and 49.97 (br, dppe). ESI-MS: calcd for ([**1**(NCMe)]$^{2+}$–MeCN), 378.0562; found, 378.0558. Anal. Calcd. for $C_{41}H_{48}NP_2S_2B_2F_8CoNi$: C, 50.66; H, 4.98. Found: C, 51.02; H, 5.15.

H_2 splitting. In a Schlenk flask, [**1**(NCMe)]$^{2+}$ (20 mg, 0.02 mmol) was dissolved in 15 mL of MeCN, the solution changed to dark brown after bubbling hydrogen (1 atm) for 5 min. The CH_3OH solution of CH_3ONa (1 mg, 0.02 mmol) was then added. the solvent was removed under reduced pressure and the product was washed with 3×10 mL of Et_2O. Yield: 15 mg (89 %). ^{1}H NMR (CD_2Cl_2): δ 1.49 (s, 15H, C_5H_{15}), −9.77 (1H, s, Co-*H*). ^{31}P{^{1}H} NMR (CD_2Cl_2): δ 67.52.

Electrochemical H_2 production. A solution of [**1**H]$^+$ (4.5 mg, 0.005 mmol) in 5 mL of MeCN was prepared in the CV cell, and was treated with successive equivalent of $Cl_2CHCOOH$. For blank experiment, a solution of acid in 5 mL of MeCN was recorded with same sweep speed of 100 mV/s.

3. Figures

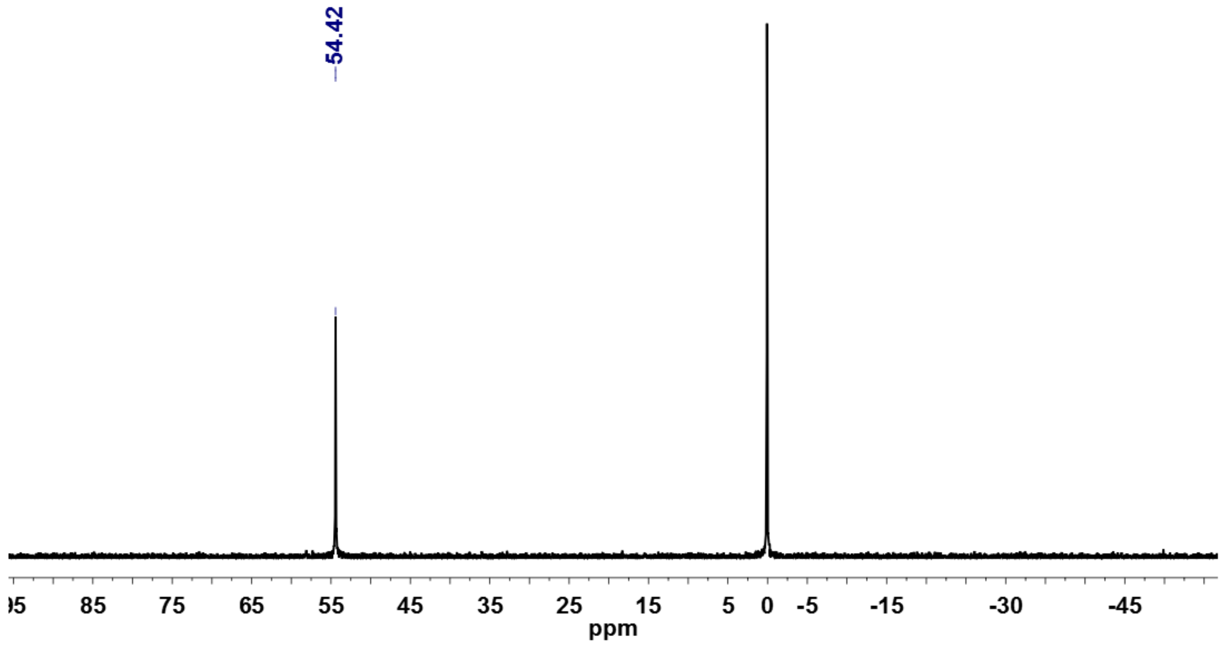

Fig. S1 $^{31}P\{^{1}H\}$ NMR spectrum of [(dppe)Ni(pdt)(Cl)CoCp*]$^+$, [**1**Cl]$^+$ in CD_3CN. (H_3PO_4 as internal standard)

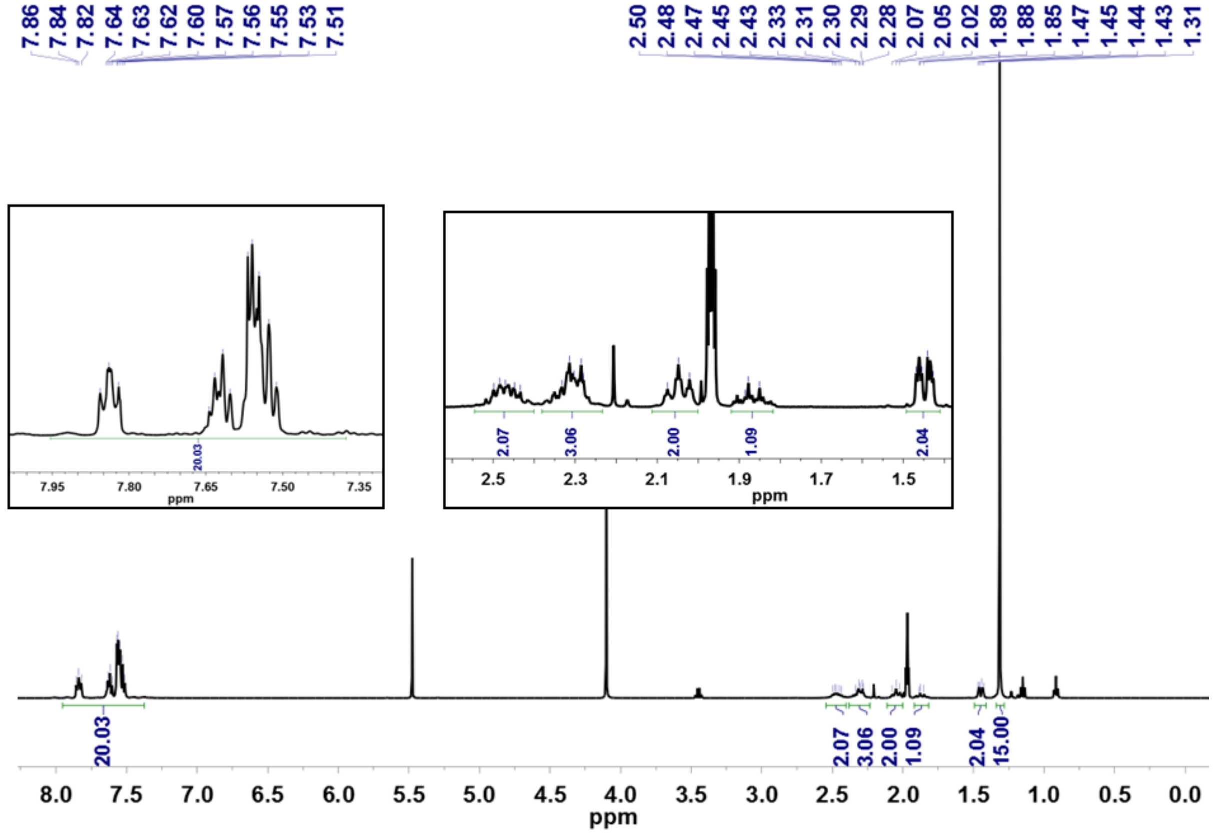

Fig. S2 ^{1}H NMR spectrum of [(dppe)Ni(pdt)(Cl)CoCp*]$^{+}$, [**1**Cl]$^{+}$ in CD_3CN. Resonances at 5.4 (CH_2Cl_2); 4.2 (H_3PO_4); 2.2 (H_2O); 1.97 (CH_3CN); 1.28, 0.89 (hexane) are from solvent.

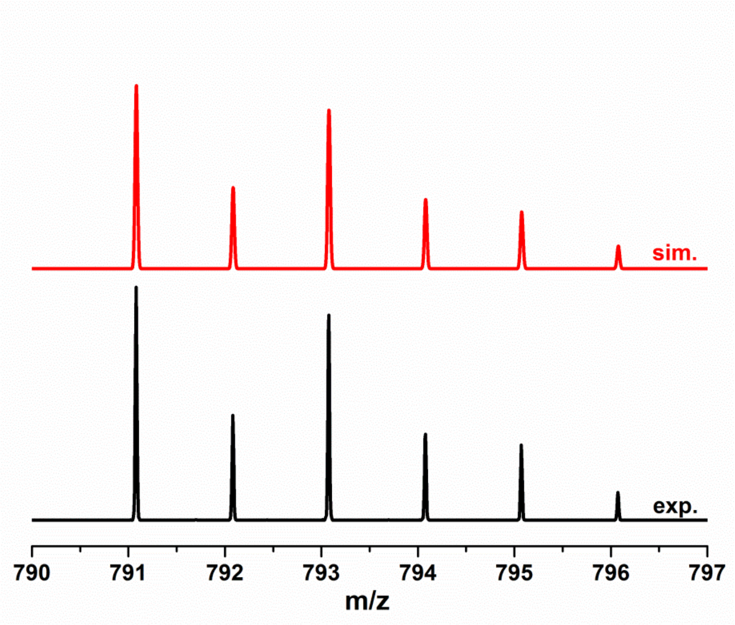

Fig. S3 ESI-MS of [(dppe)Ni(pdt)(Cl)CoCp*]$^{+}$, [**1**Cl]$^{+}$.

Result: Calcd for [**1**Cl]$^{+}$, 791.0812; found, 791.0796.

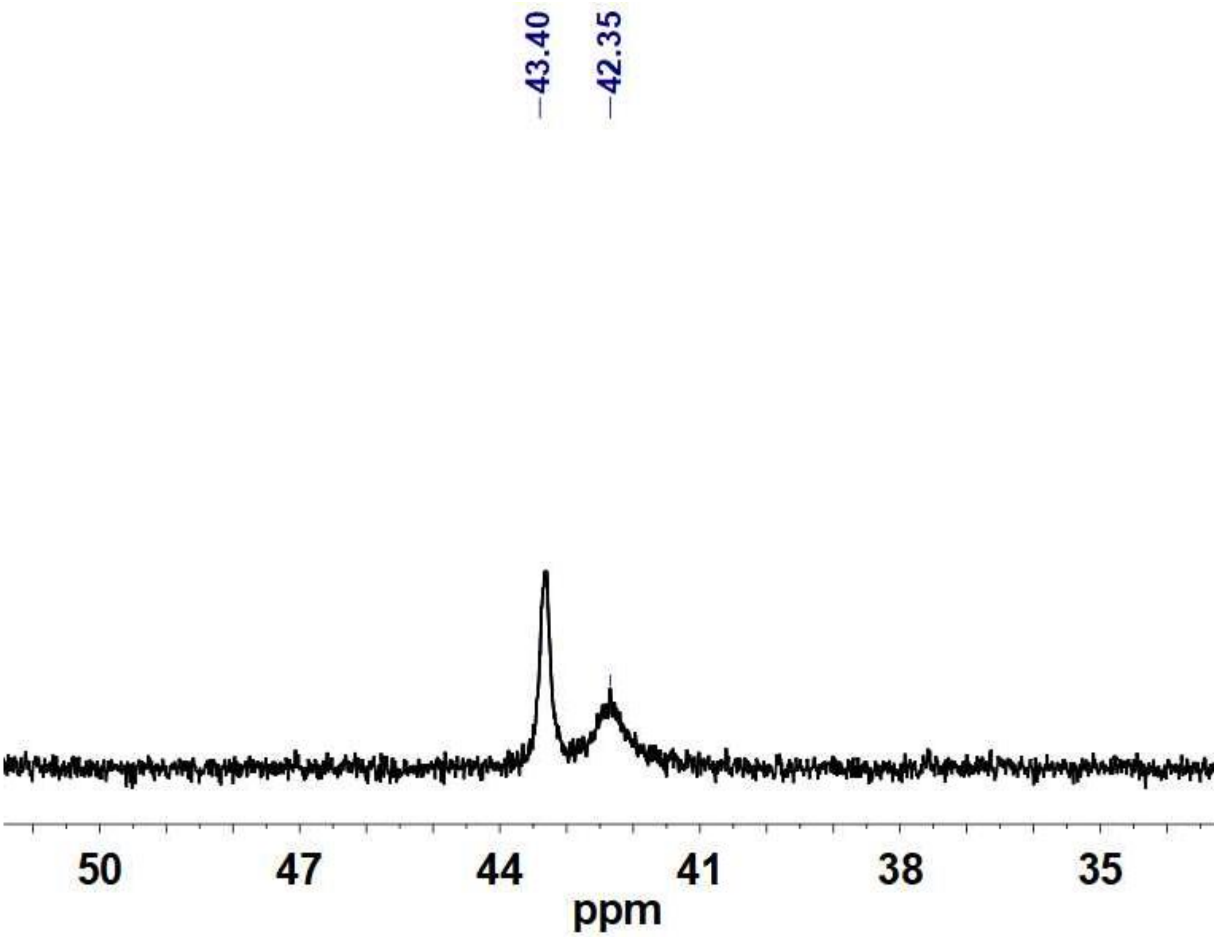

Fig. S4 ^{31}P NMR spectrum of [(dppe)Ni(pdt)CoCp*], **1** in CD_2Cl_2. The signal of H_3PO_4 is at 0 ppm.

Result: The signal at 43.40 ppm is for [$Ni^{I}Co^{II}$] species and the signal at 42.35 ppm is for [$Ni^{II}Co^{I}$] species.

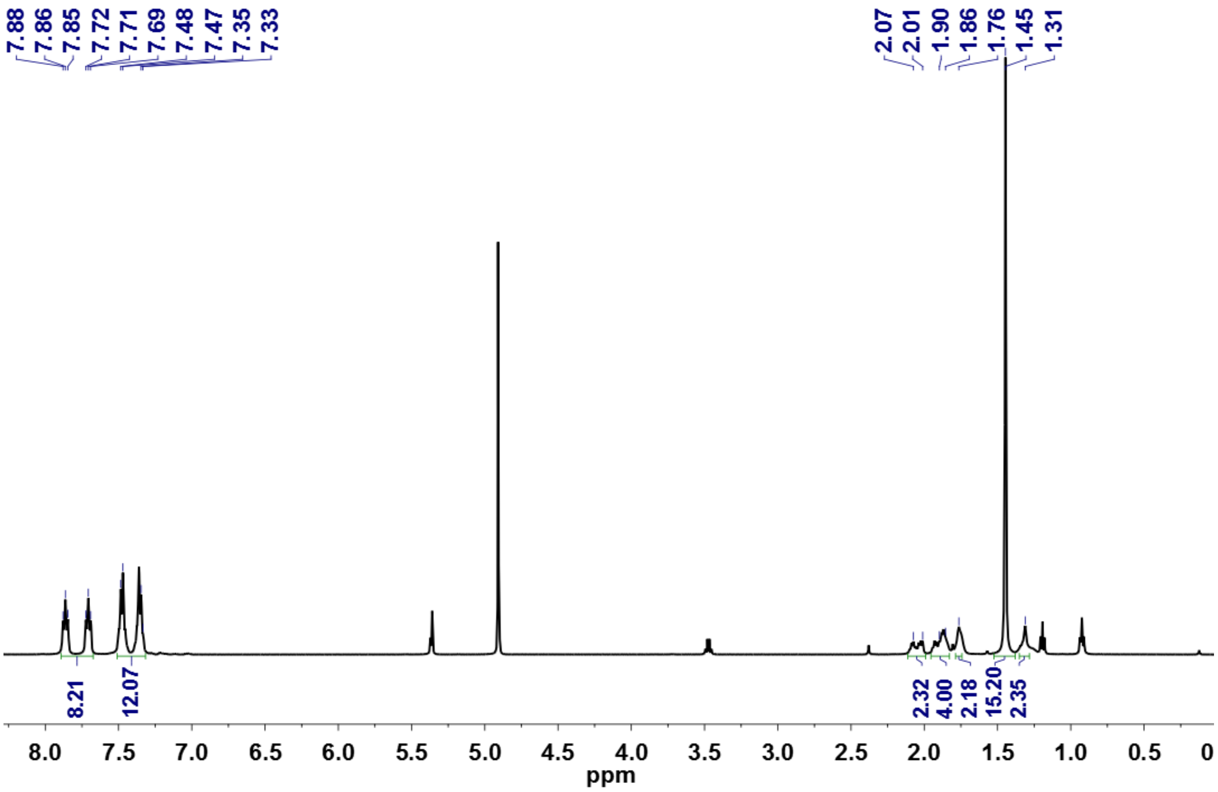

Fig. S5 ^{1}H NMR spectrum of [(dppe)Ni(pdt)CoCp*], **1** in CD_2Cl_2. Resonances at 5.4 (CH_2Cl_2); 4.7 (H_2O); 3.43, 1.15 (Et_2O); 1.28, 0.89 (hexane) are from solvent.

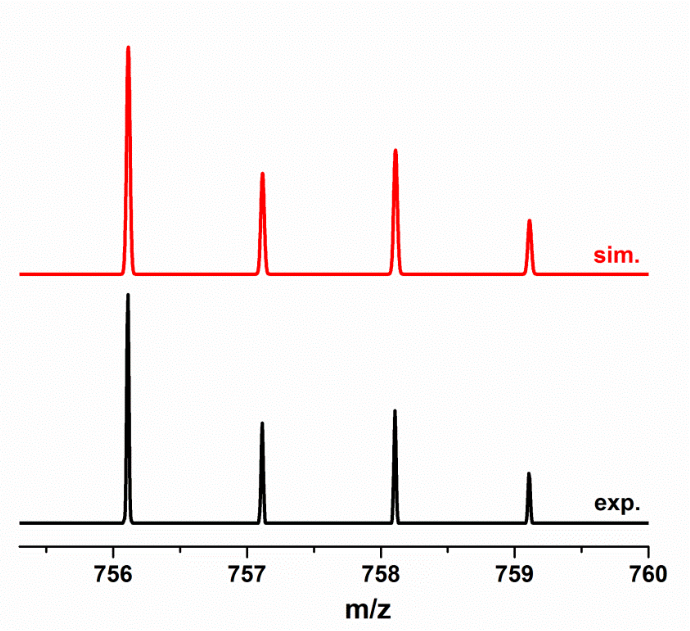

Fig. S6 ESI-MS of [(dppe)Ni(pdt)CoCp*], **1**.

Result: Calcd for 1, 756.1123; found, 756.1090.

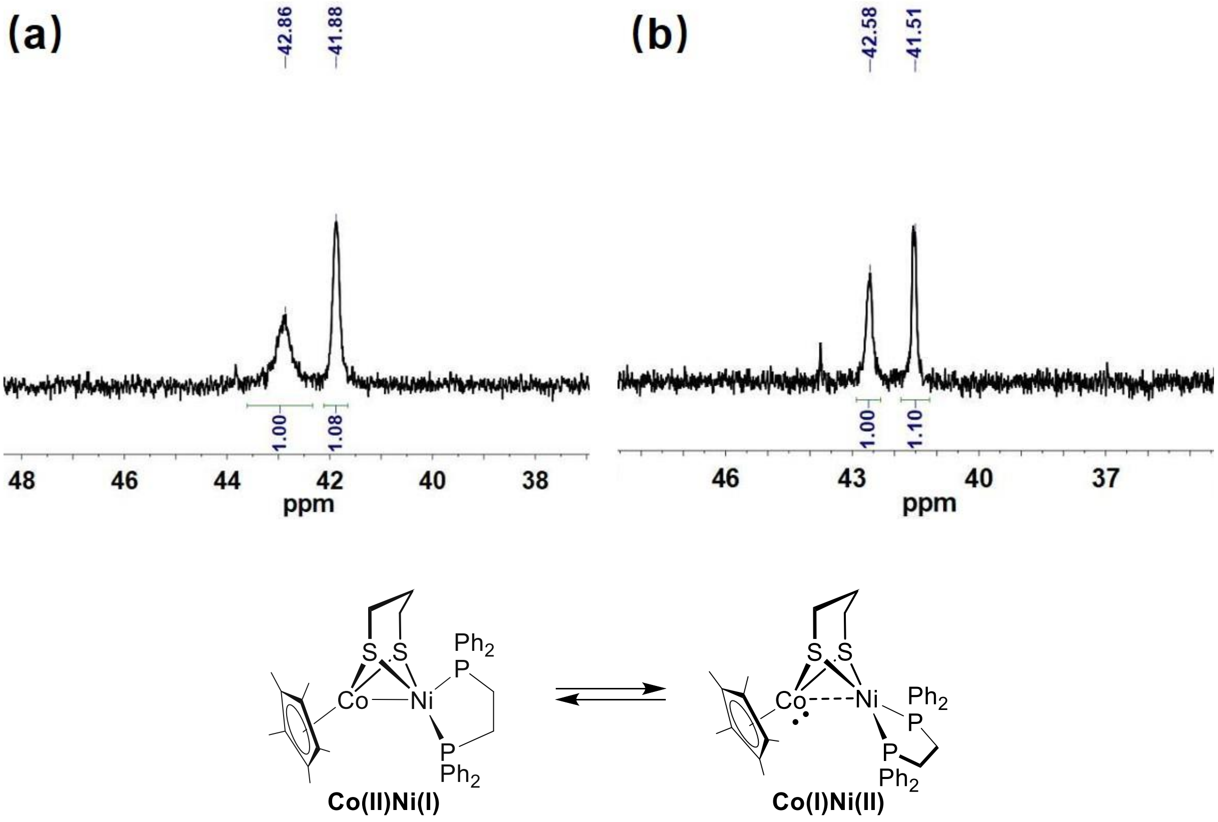

$$K_{eq} = \frac{[Co(I)Ni(II)]}{[Co(II)Ni(I)]} \approx 1$$

Fig. S7 ^{31}P NMR spectrum of [(dppe)Ni(pdt)CoCp*], **1** in benzonitrile at (a) 298 K and (b) 323 K.

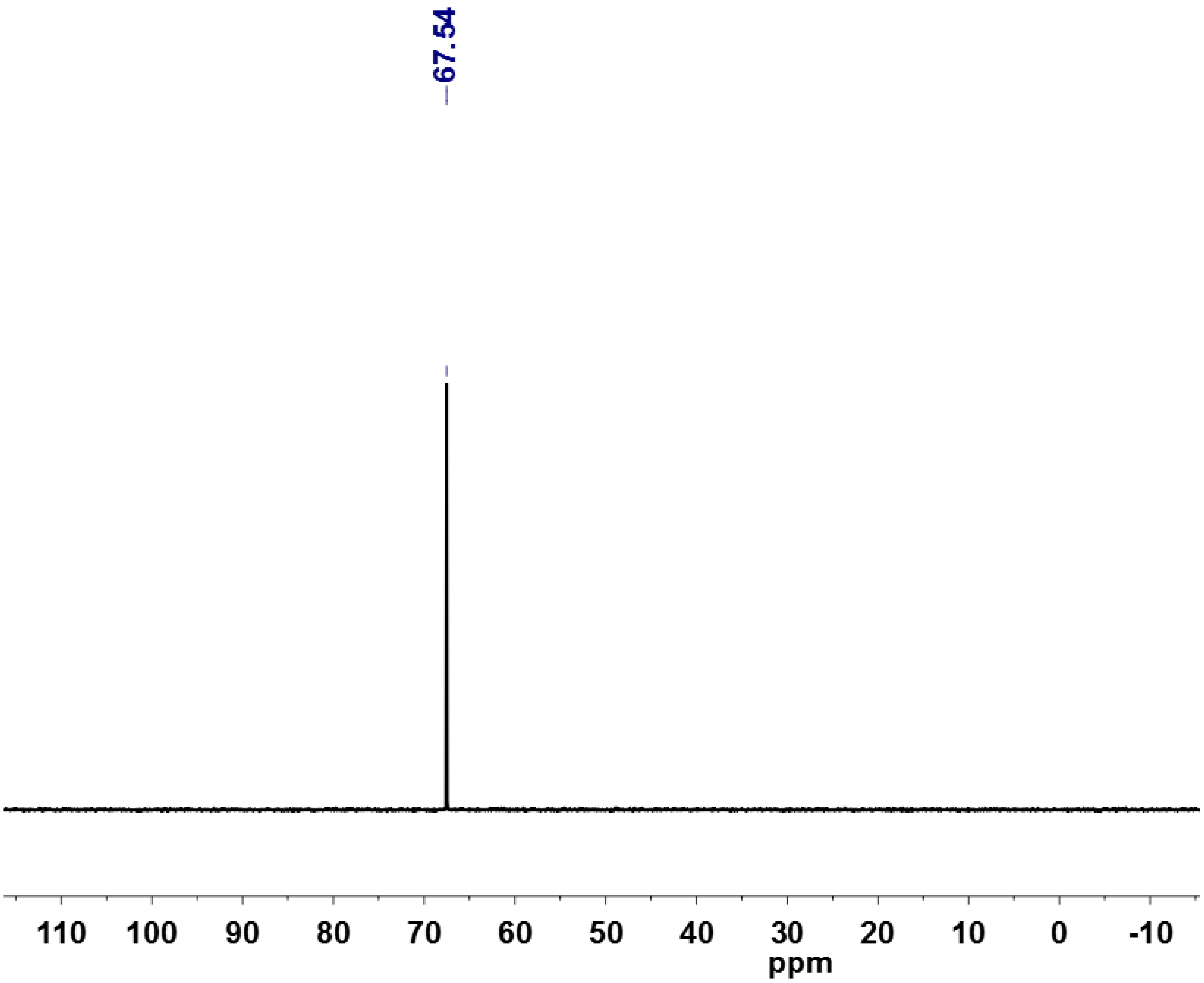

Fig. S8 $^{31}P\{^1H\}$ NMR spectrum of [(dppe)Ni(pdt)(H)CoCp*]$^+$, [**1**H]$^+$ in CD_2Cl_2.

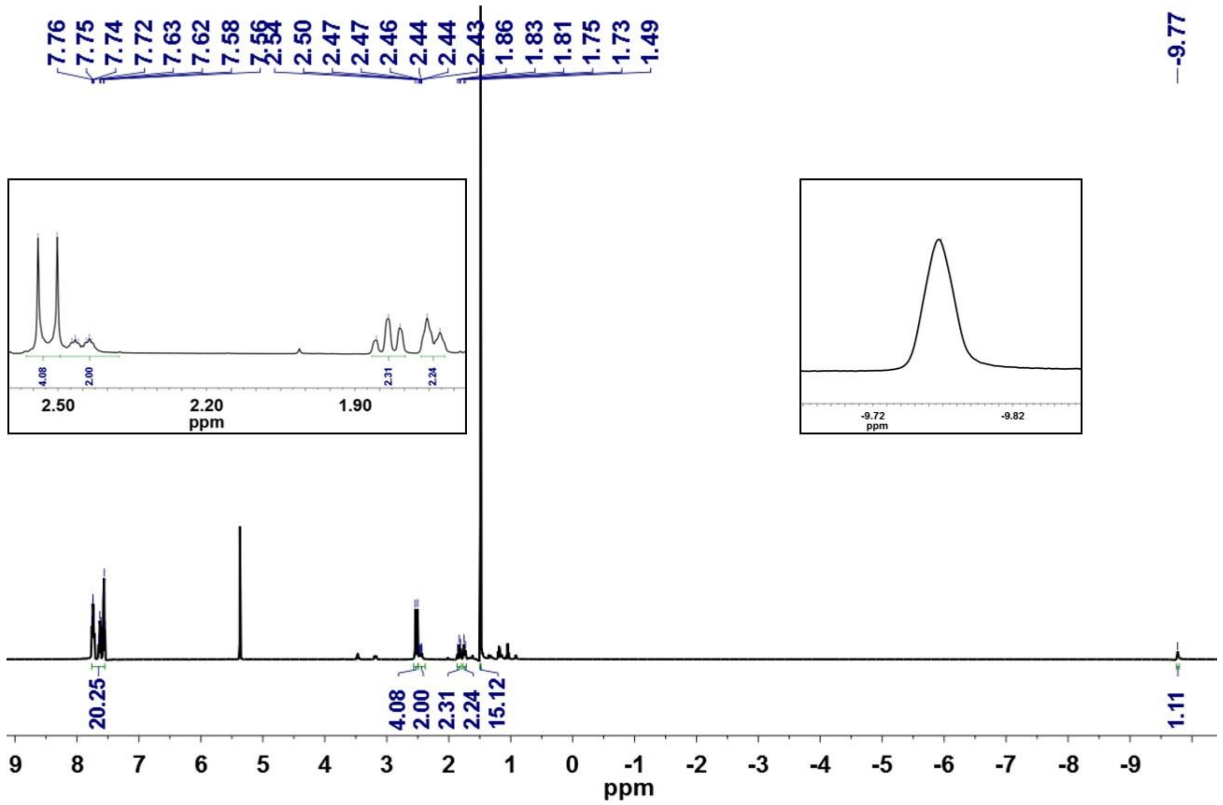

Fig. S9 ^{1}H NMR spectrum of [(dppe)Ni(pdt)(H)CoCp*]$^{+}$, [**1**H]$^{+}$ in CD_2Cl_2. Resonances at 5.4 (CH_2Cl_2); 1.28, 0.89 (hexane) are from solvent.

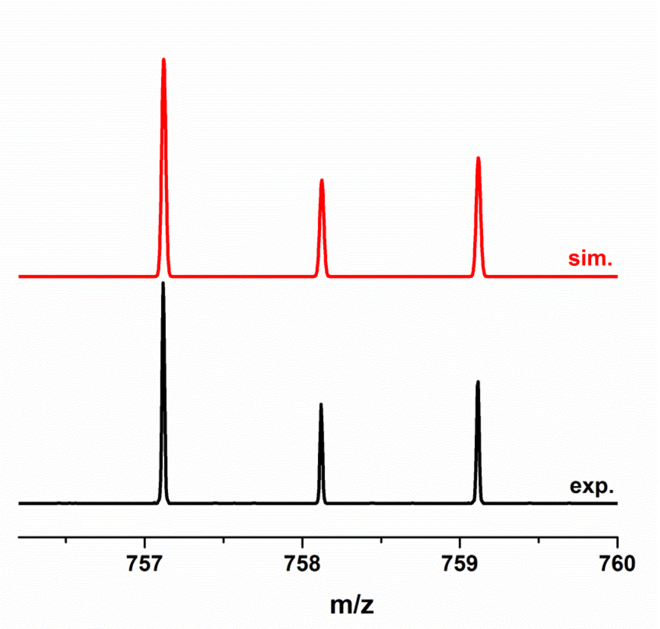

Fig. S10 ESI-MS of [(dppe)Ni(pdt)(H)CoCp*]$^+$, [**1**H]$^+$.

Result: Calcd for [**1**H]$^+$, 757.1202; found, 757.1175.

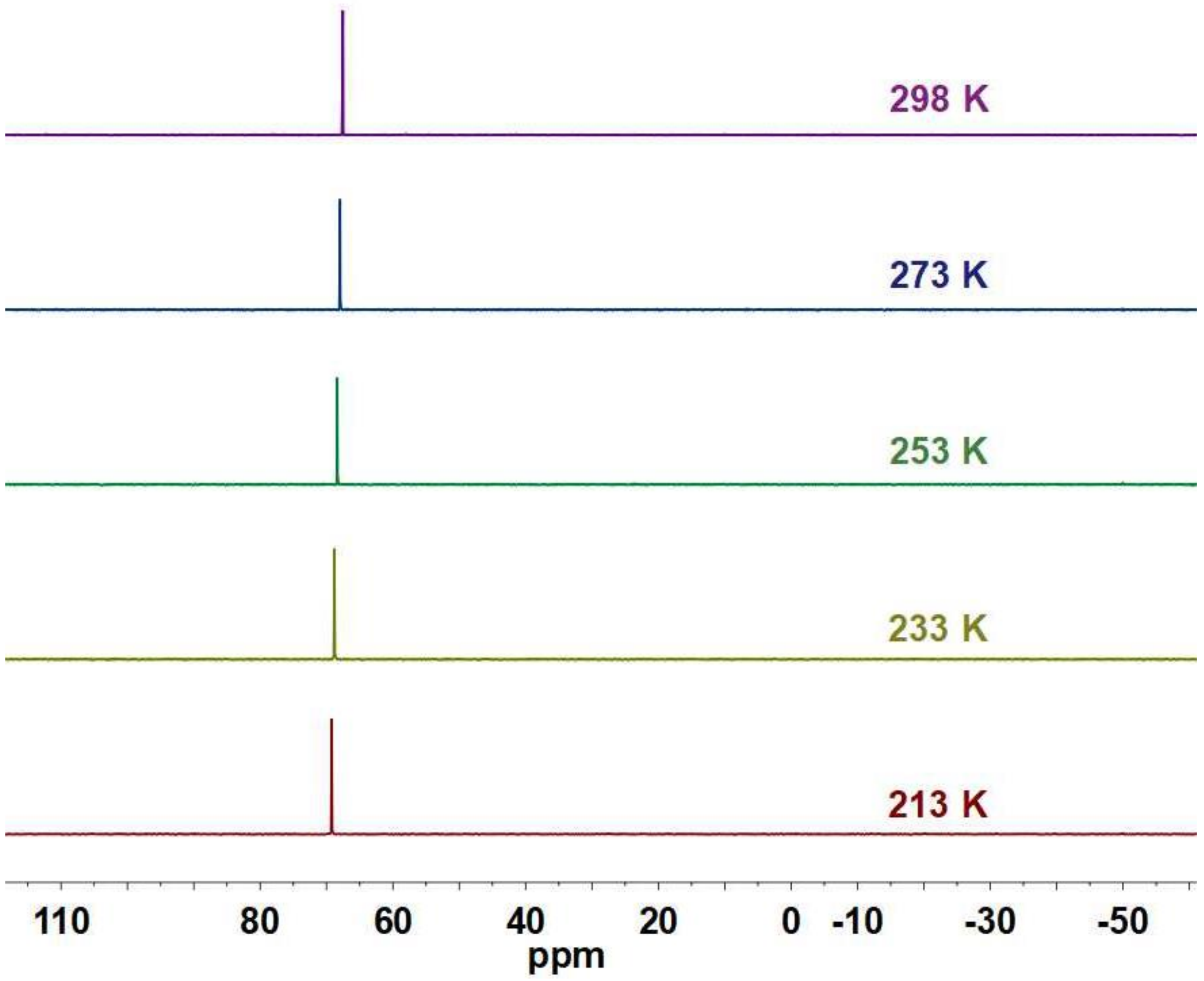

Fig. S11 $^{31}P\{^1H\}$ NMR spectrum of [(dppe)Ni(pdt)(H)CoCp*]$^+$, [**1**H]$^+$ at various temperatures in CD_2Cl_2.

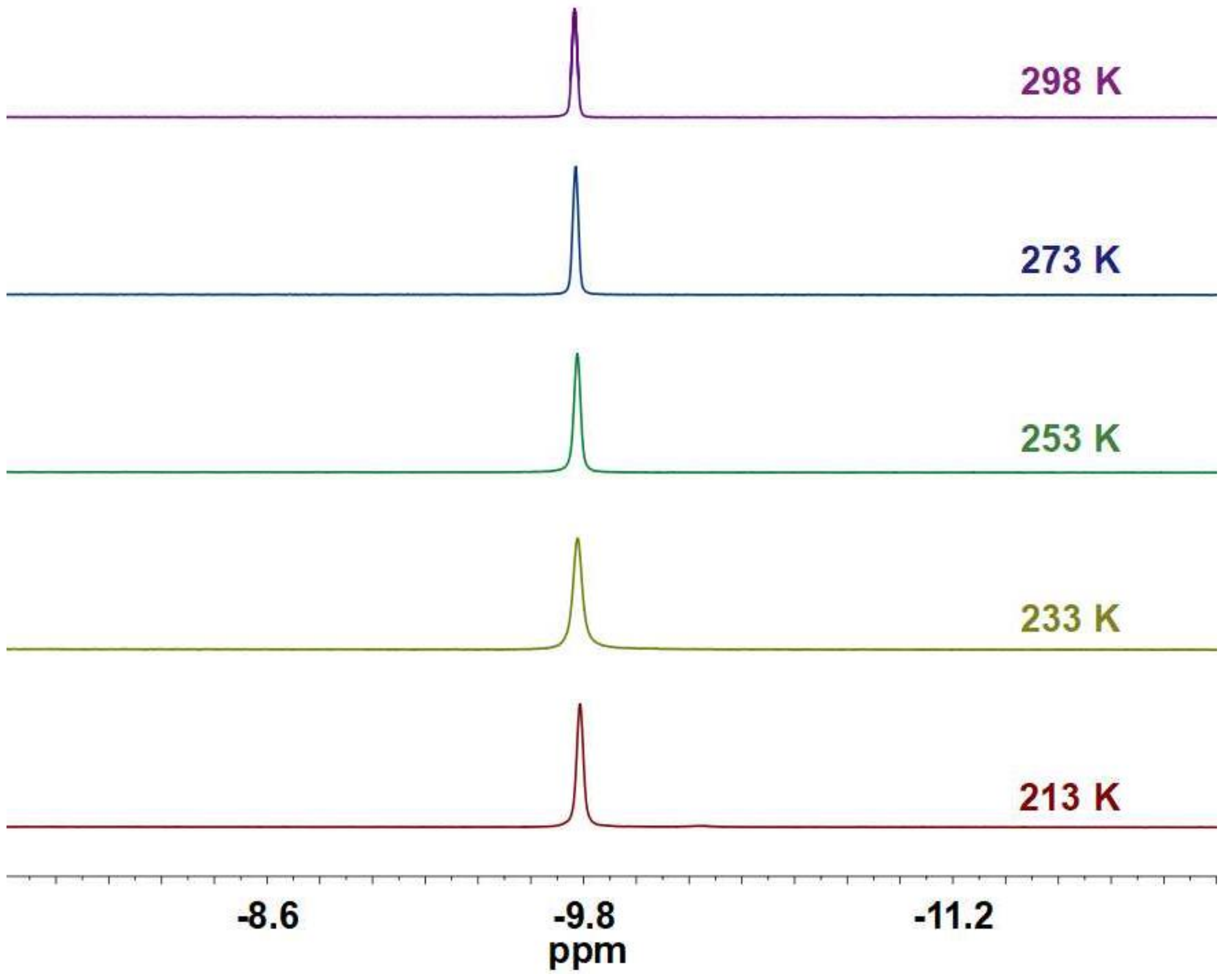

Fig. S12 ^{1}H NMR spectrum of [(dppe)Ni(pdt)(H)CoCp*]$^{+}$, [**1**H]$^{+}$ at various temperatures in CD_2Cl_2.

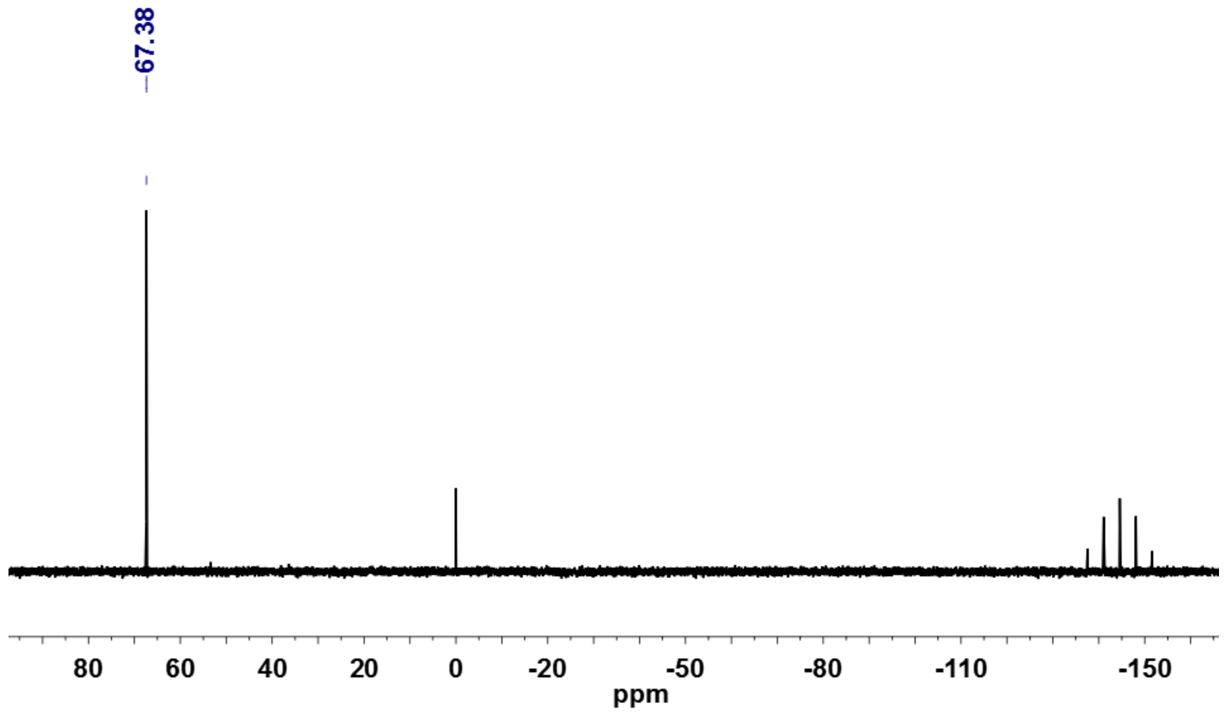

Fig. S13 $^{31}P\{^1H\}$ NMR spectrum of [(dppe)Ni(pdt)(D)CoCp*]$^+$, [**1**D]$^+$ in CH_2Cl_2. (H_3PO_4 as internal standard).

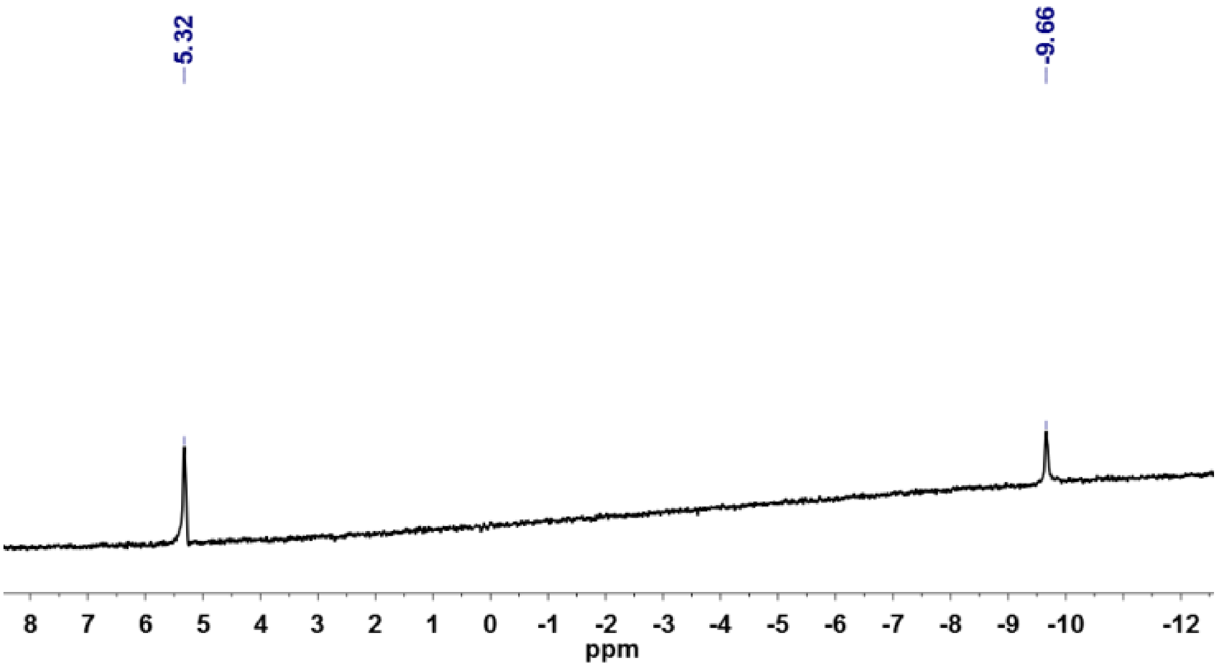

Fig. S14 ^{2}H NMR spectrum of [(dppe)Ni(pdt)(D)CoCp*]$^{+}$, [**1**D]$^{+}$ in CH_2Cl_2.

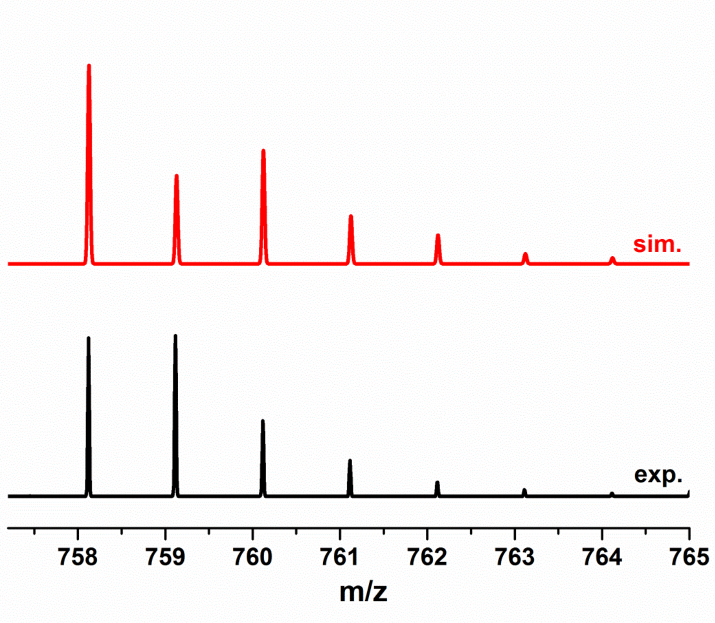

Fig. S15 ESI-MS of [(dppe)Ni(pdt)(D)CoCp*]$^{+}$, [**1**D]$^{+}$.

Result: Calcd for [**1**D]$^{+}$, 758.1264; found, 758.1216.

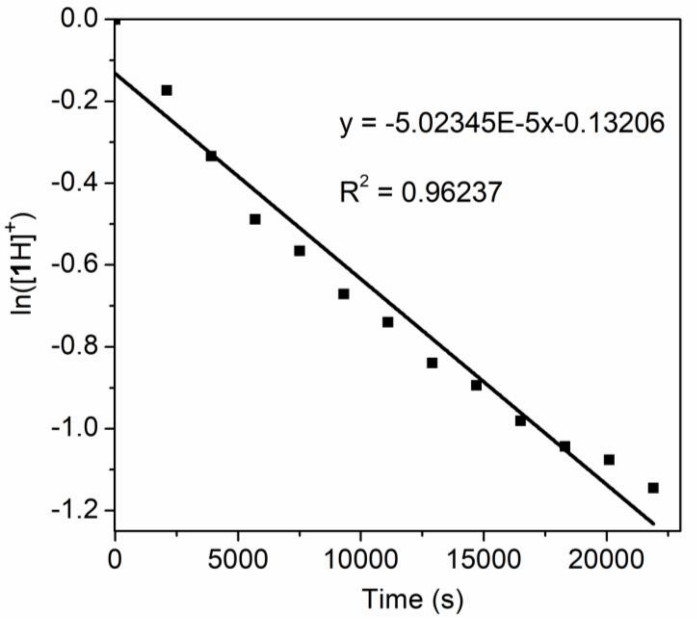

Fig. S16 Plot of the hydride integration vs time for the reaction of [**1**H]$^+$ with excess D_2O in CD_3CN.

Results: k = 5.023 x 10^{-5} s^{-1}, $t_{1/2}$ = 3.83 h.

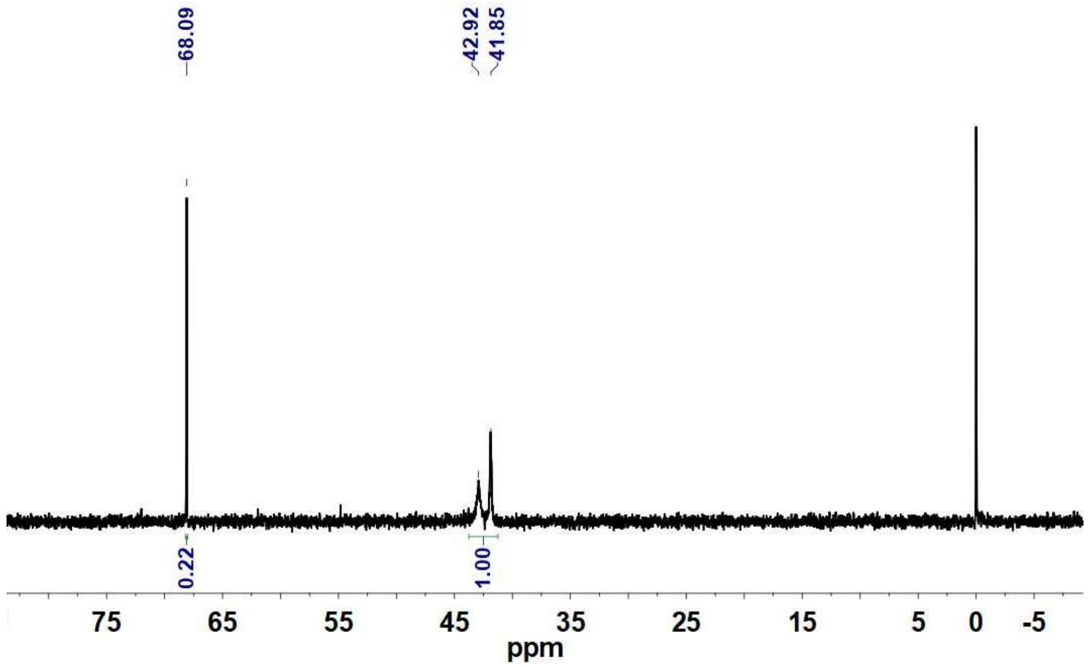

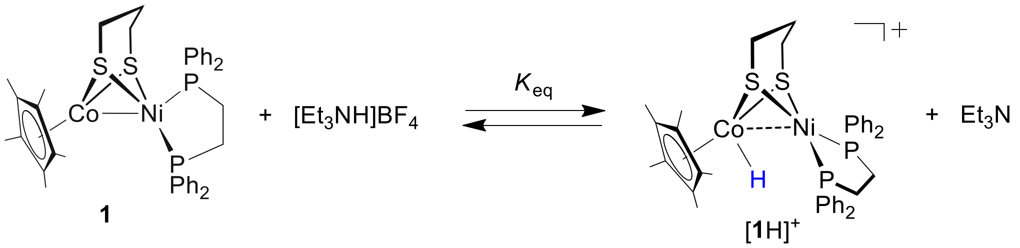

$$K_{eq} = \frac{[[\mathbf{1}\text{H}]^+][\text{Et}_3\text{N}]}{[\mathbf{1}][\text{Et}_3\text{NH}^+]} = \frac{K_c(\text{H}[\text{Et}_3\text{N}]^+)}{K_c([\mathbf{1}\text{H}]^+)}$$

$$\log K_{eq} = \log K_c([\text{Et}_3\text{NH}]^+) - \log K_c([\mathbf{1}\text{H}]^+)$$
$$= -\text{p}K_a([\text{Et}_3\text{NH}]^+) - -\text{p}K_a([\mathbf{1}\text{H}]^+)$$

$$\text{p}K_a([\mathbf{1}\text{H}]^+) = \text{p}K_a([\text{Et}_3\text{NH}]^+) + \log K_{eq}$$
$$= 18.8 + \log K_c$$
$$= 17.5 \pm 0.1$$

Fig. S17 ^{31}P{^{1}H} NMR spectrum of **1** with $[Et_3NH]BF_4$ in PhCN up to the balance.

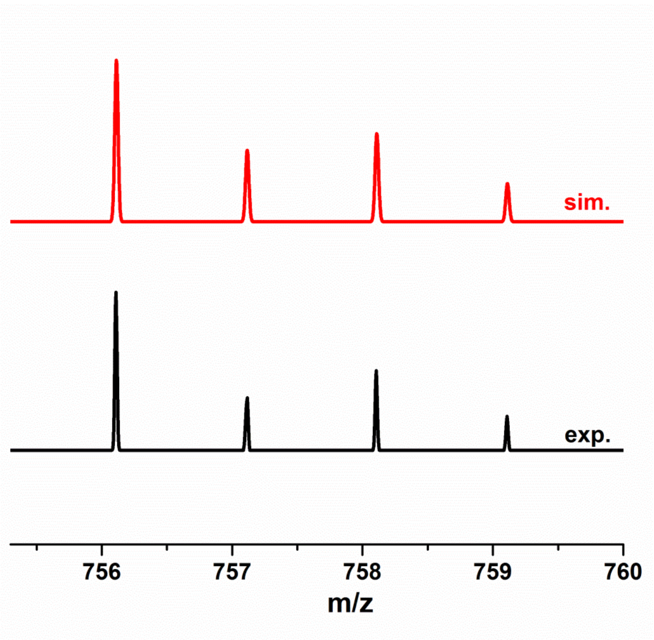

Fig. S18 ESI-MS of [(dppe)Ni(pdt)CoCp*]$^{+}$, [**1**]$^{+}$.

Result: Calcd for [**1**]$^{+}$, 756.1123; found, 756.1088.

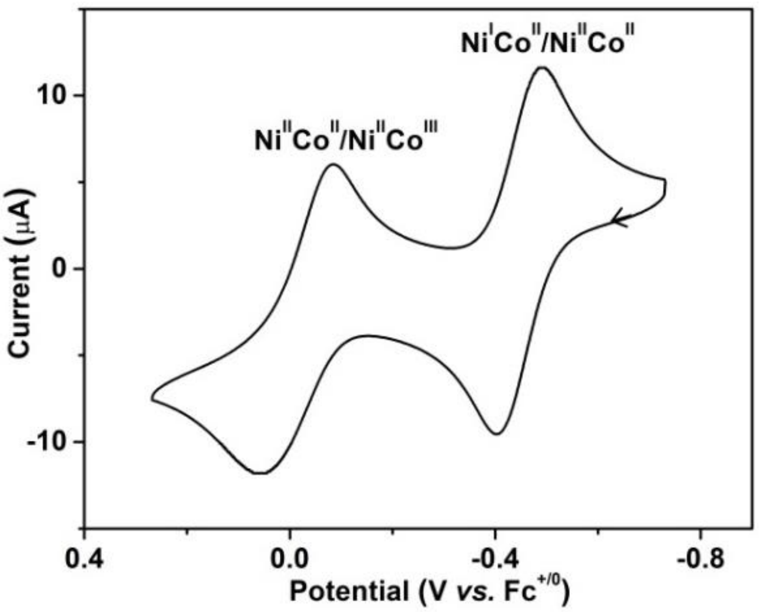

Fig. S19 Cyclic voltammogram of **1** recorded in PhCN with 0.1 M NBu_4PF_6, scan rate 100 mV/s.

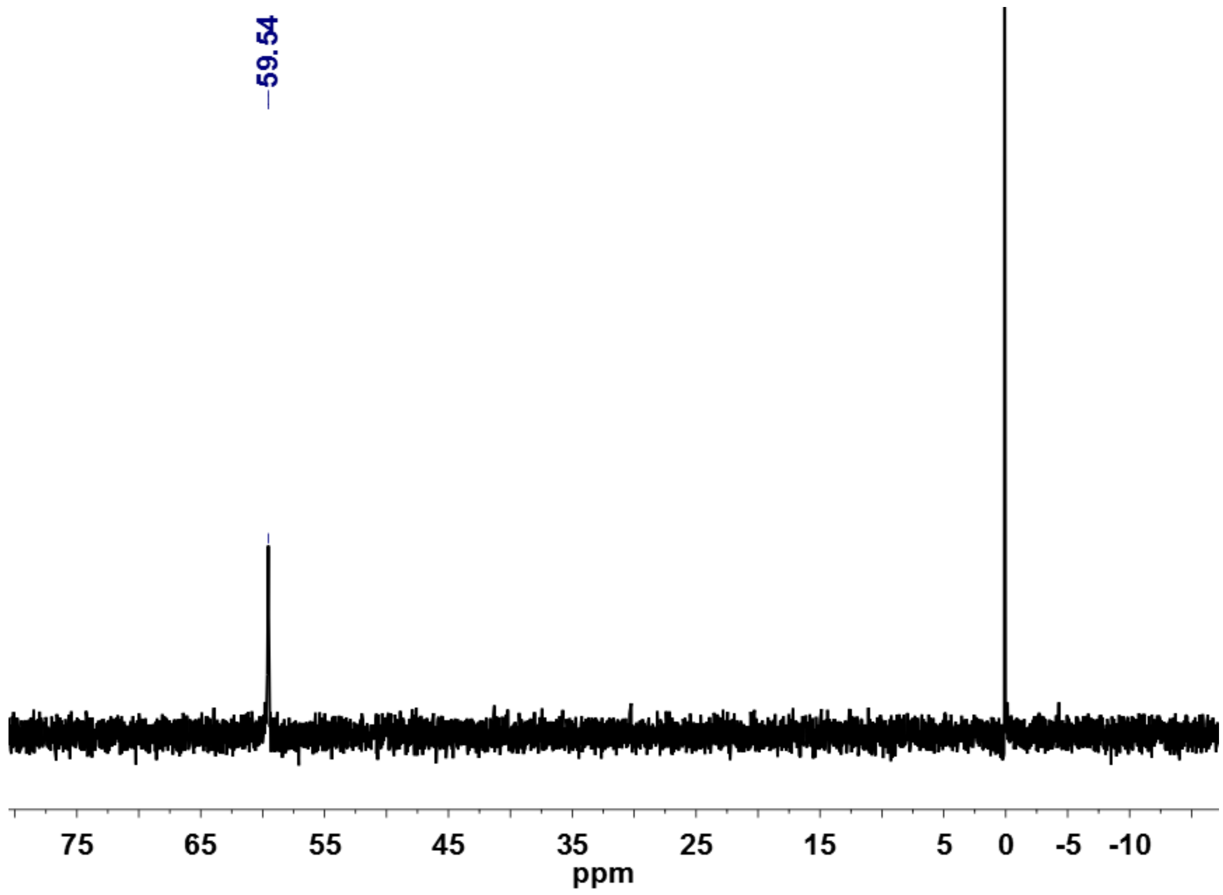

Fig. S20 ^{31}P NMR spectrum of [(dppe)Ni(pdt)CoCp*]$^{2+}$, [**1**]$^{2+}$ in CH_2Cl_2.

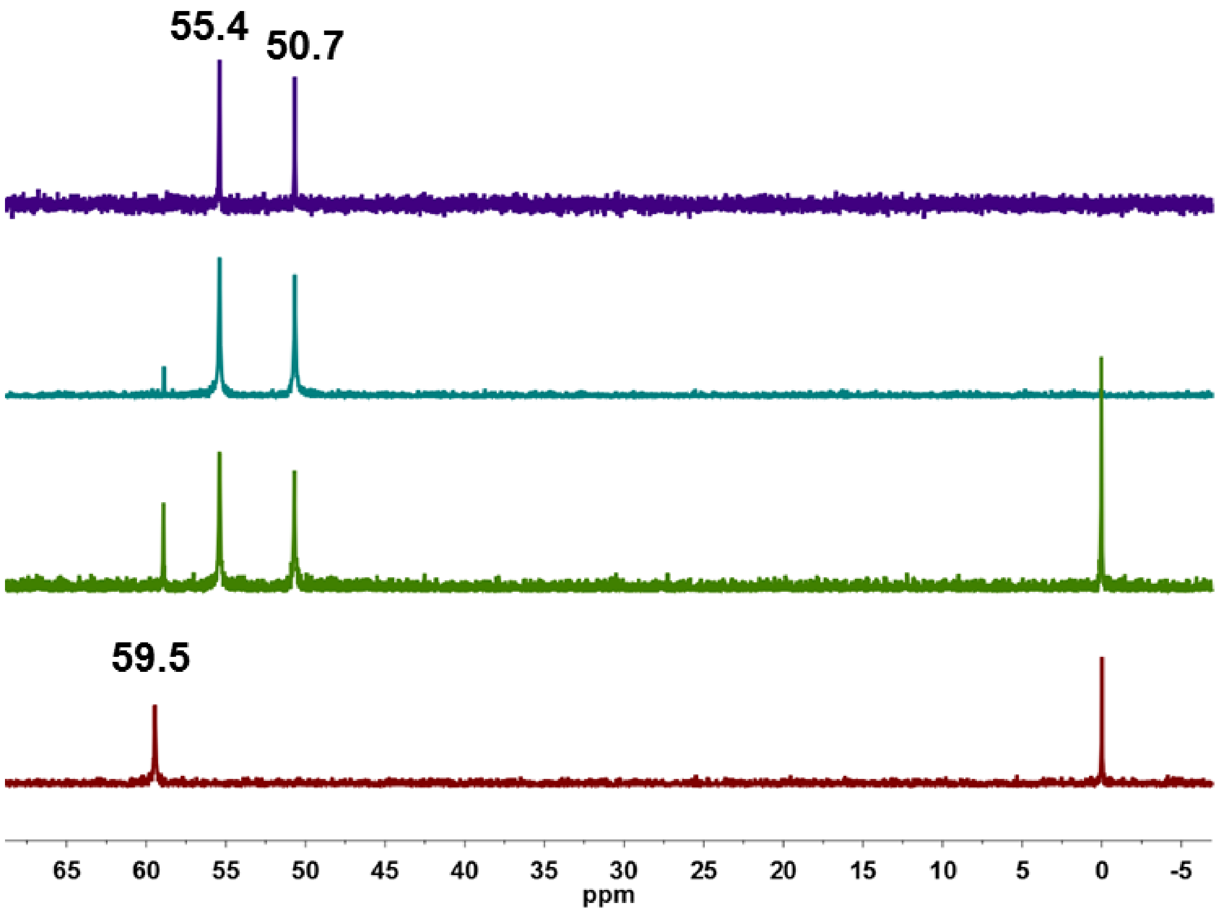

Fig. S21 ^{31}P NMR spectrum of [**1**]$^{2+}$ (red) and [**1**(NCMe)]$^{2+}$ (purple) in acetonitrile-d_3.

Results: 54.69, 49.98 ppm.

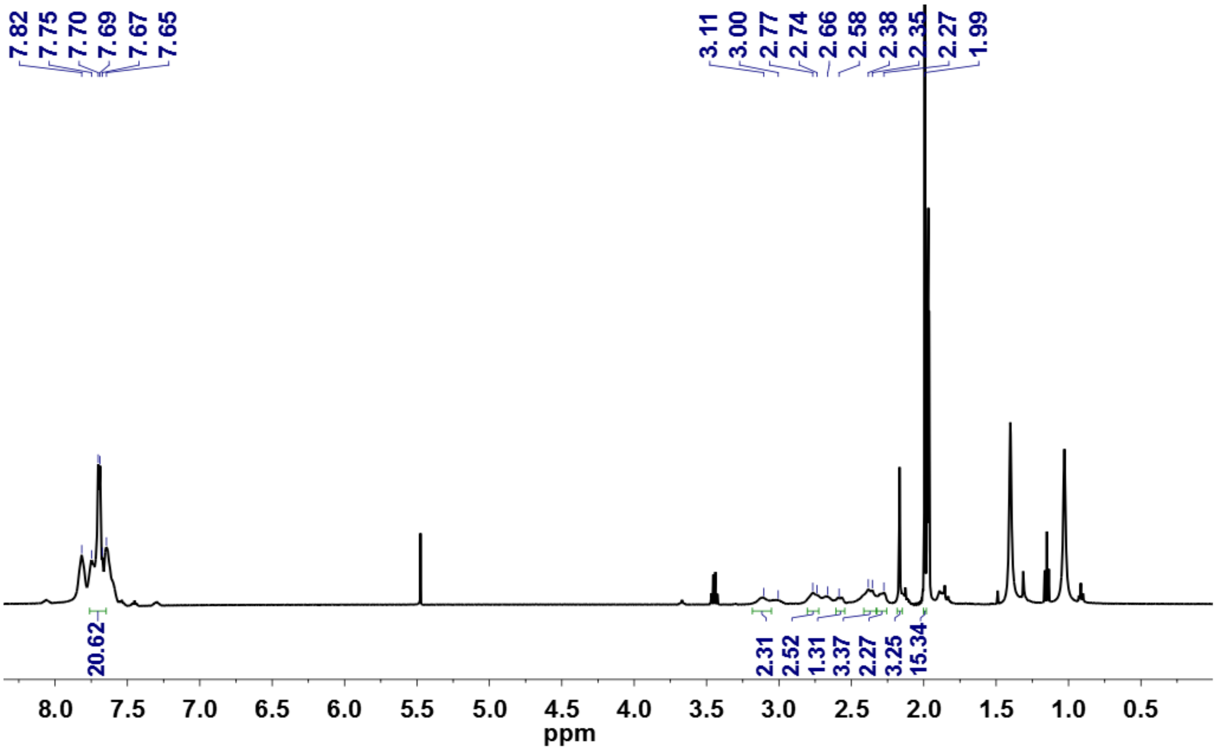

Fig. S22 ^{1}H NMR spectrum of [(dppe)Ni(pdt)Co(NCMe)Cp*]$^{2+}$, [**1**(NCMe)]$^{2+}$ in acetonitrile-d_3.

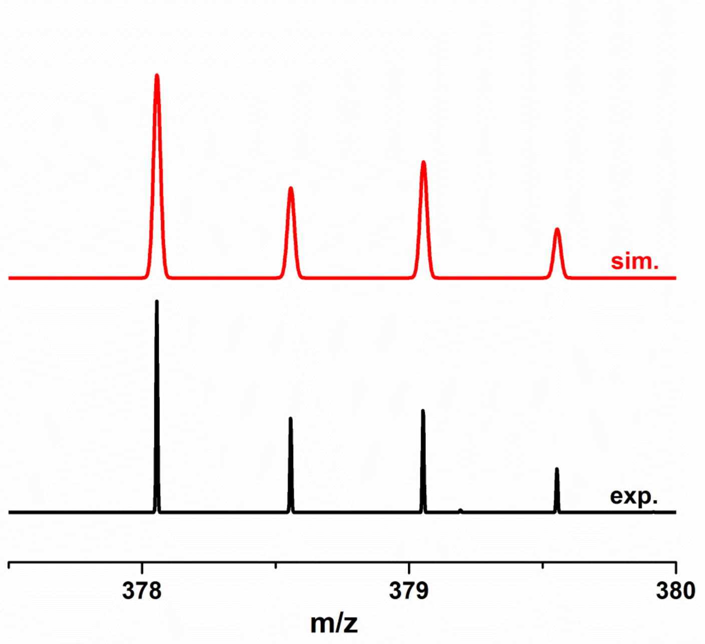

Fig. S23 ESI-MS of [(dppe)Ni(pdt)Co(NCMe)Cp*]$^{2+}$, [**1**(NCMe)]$^{2+}$.

Results: Calcd for ([**1**(NCMe)]$^{2+}$–MeCN), 378.0562; found, 378.0558.

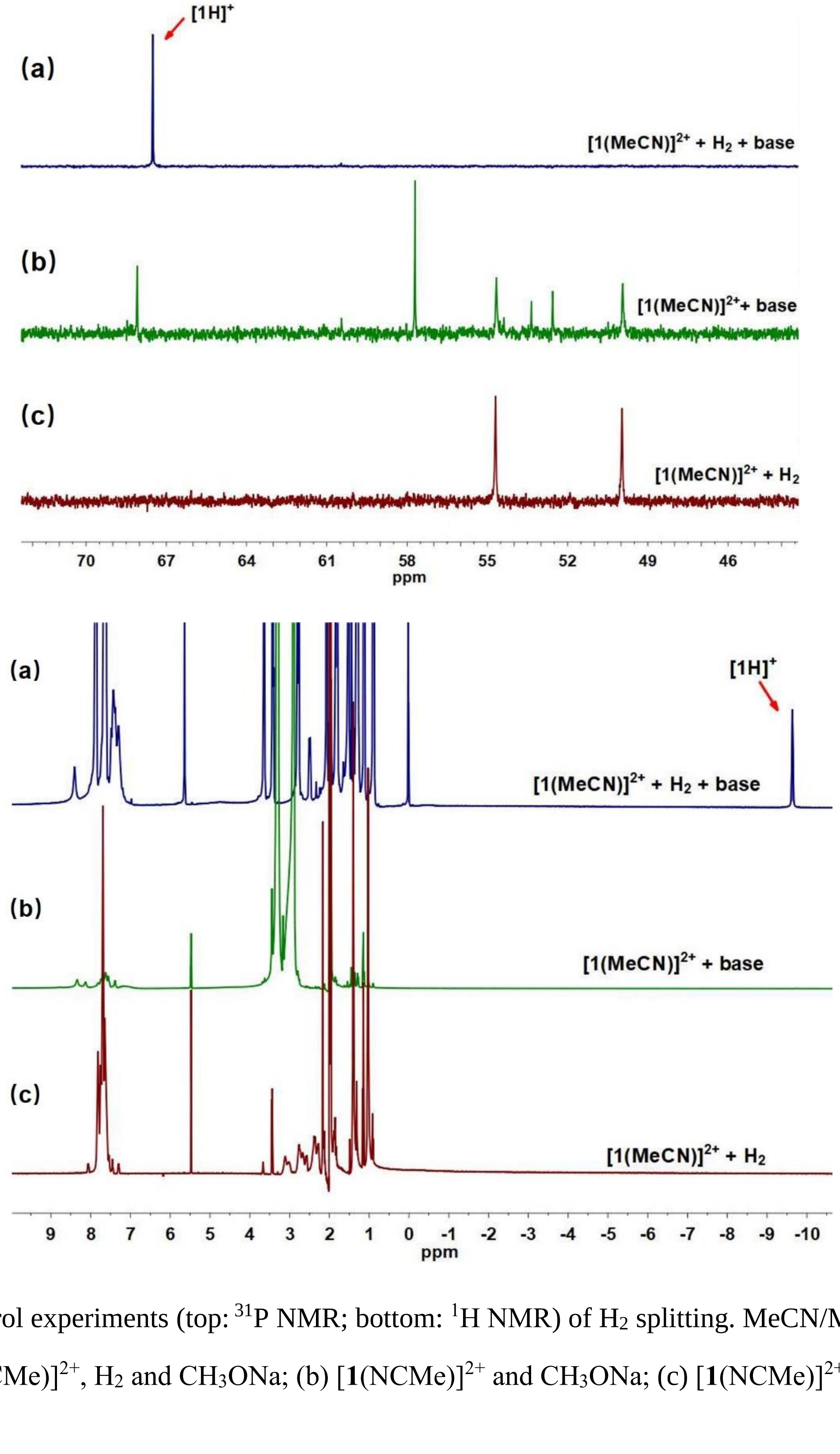

Fig. S24 Control experiments (top: ^{31}P NMR; bottom: ^{1}H NMR) of H_2 splitting. MeCN/MeOH (v:v = 1:1), (a) [**1**(NCMe)]$^{2+}$, H_2 and CH_3ONa; (b) [**1**(NCMe)]$^{2+}$ and CH_3ONa; (c) [**1**(NCMe)]$^{2+}$ and H_2.

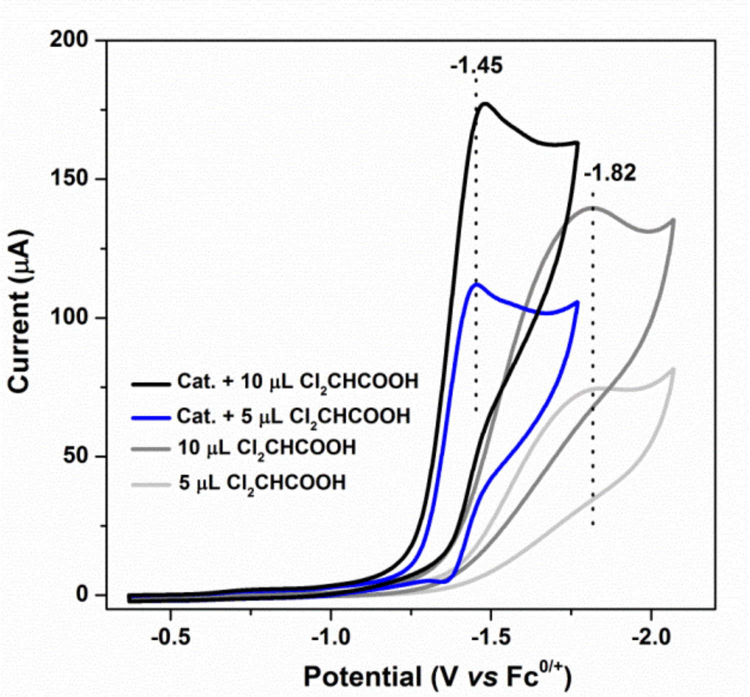

Fig. S25 Comparison of cyclic voltammograms in $Cl_2CHCOOH$ solution before and after the addition of [**1**H]$^+$. Conditions: 1 mM sample in CH_3CN, 0.1 M *n*-NBu_4PF_6 as the supporting electrolyte; scan rate = 100 mV/s.

Table S1. Selected parameters of 1 mM [**1**H]$^+$ in MeCN in addition of $Cl_2CHCOOH$.

Acid (μL)	i_{cat}/i_p	TOF (s^{-1})	$E_{cat1/2}$ (V)	Overpotential (V)
1	1.416	0.389	-1.325	0.512
2	1.622	0.510	-1.337	0.515
4	2.717	1.432	-1.342	0.511
6	3.377	2.212	-1.353	0.517
8	3.493	2.367	-1.339	0.499
10	5.366	5.586	-1.367	0.524
12	6.150	7.336	-1.377	0.532
14	7.482	10.861	-1.375	0.528
16	8.258	13.231	-1.395	0.546
18	8.786	14.977	-1.392	0.541
20	9.439	17.285	-1.407	0.555
25	11.690	26.512	-1.43	0.575
30	13.948	37.744	-1.45	0.593
35	15.317	45.511	-1.45	0.591
40	17.979	62.709	-1.48	0.619
45	19.994	77.553	-1.492	0.630
50	21.732	91.624	-1.486	0.622
55	25.209	123.289	-1.486	0.621
60	25.503	126.182	-1.506	0.640
65	27.124	142.723	-1.5	0.633
70	28.131	153.522	-1.52	0.652
75	29.324	166.823	-1.517	0.648
85	32.824	209.022	-1.535	0.665
90	35.472	244.097	-1.531	0.660

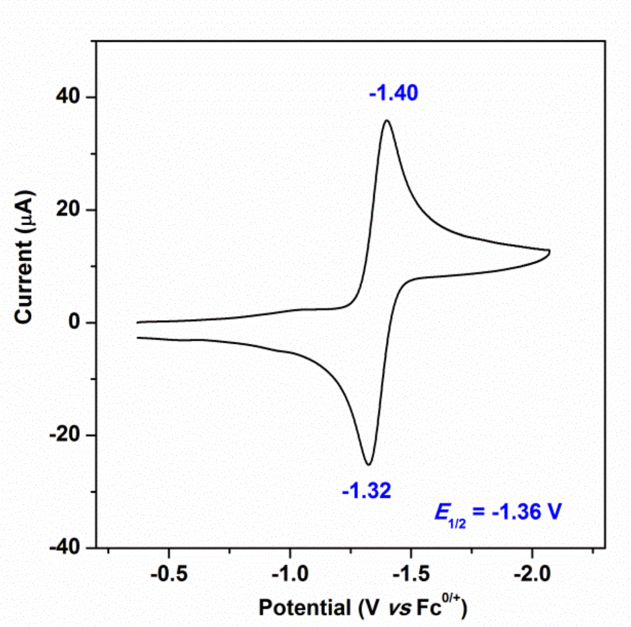

Results: The reversible redox wave is assigned to [Co^{III}HNi^{II}/Co^{II}HNi^{II}] couple, i_{pa}/i_{pc} = 0.98.

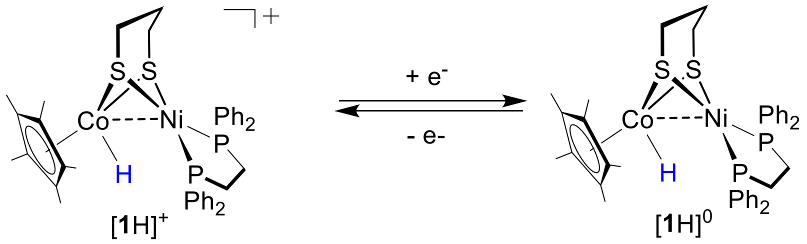

Fig. S26 Cyclic voltammogram of [**1**H]$^+$ in MeCN. Conditions: ~ 1 mM [**1**H]$^+$, 0.1 M *n*-NBu_4PF_6, scan rate = 100 mV/s.

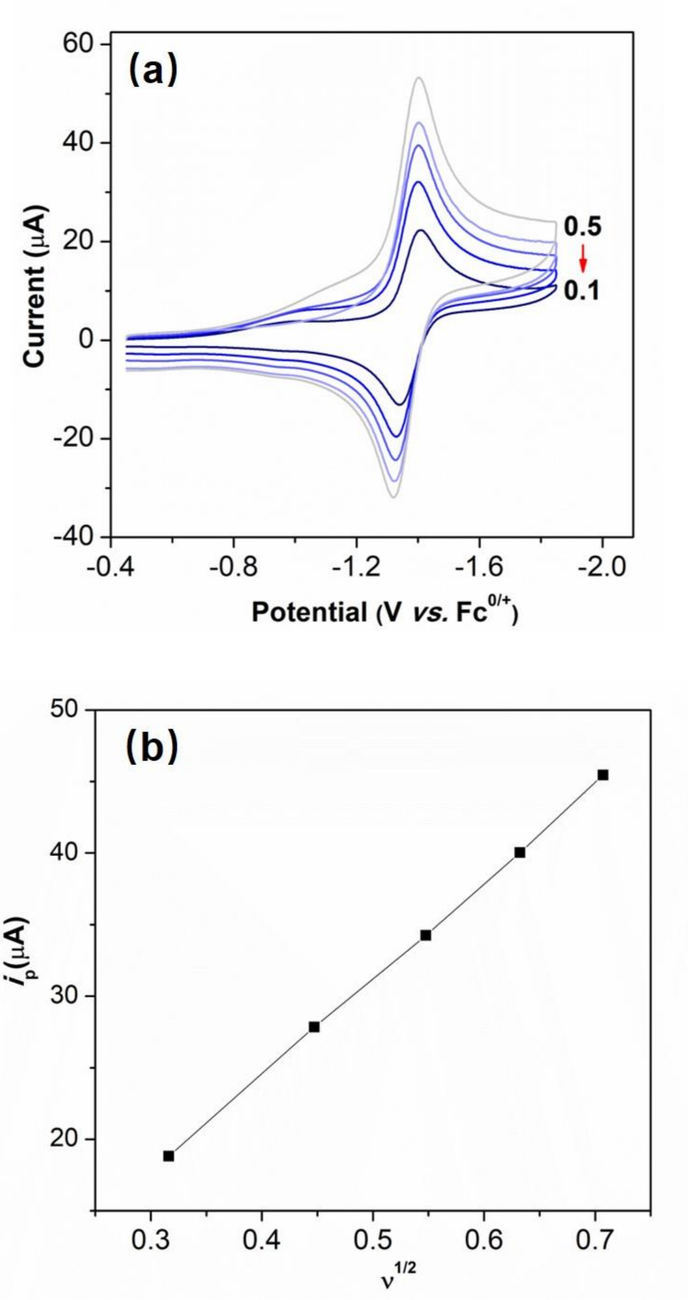

Fig. S27 (a) Cyclic voltammogram of [**1**H]$^+$ at various scan rate and (b) scan rate dependence of i_p for the couples [**1**H]$^{+/0}$ in MeCN. Conditions: ~ 0.5 mM [**1**H]$^+$, 0.1 M *n*-NBu_4PF_6, scan rate = 0.1-0.5 V/s.

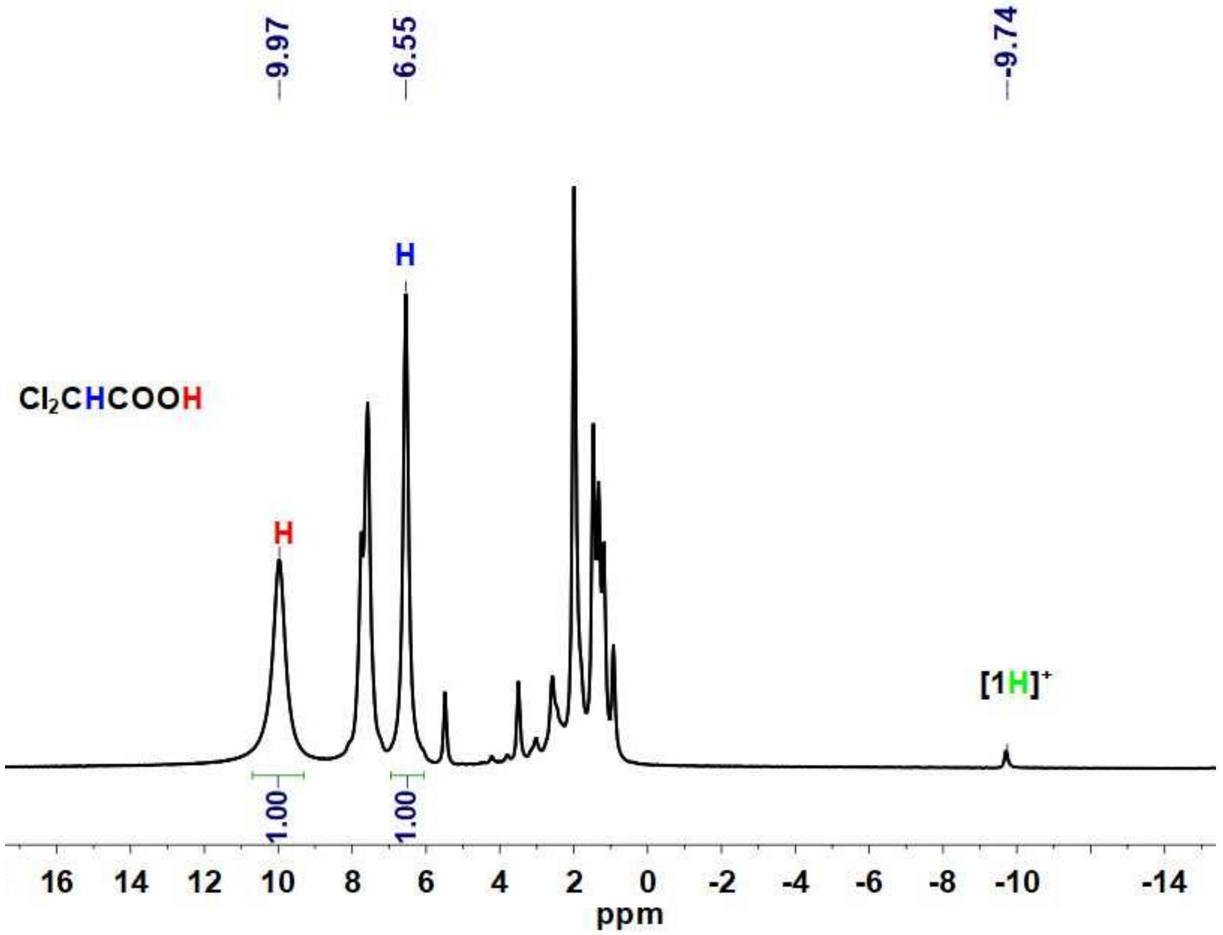

Results: [**1**H]$^+$ coexists with the excessive $Cl_2CHCOOH$ in MeCN proves that [**1**H]$^+$ is stable in acid condition for the electrochemical reaction.

Fig. S28 ^{1}H NMR spectrum of [**1**H]$^+$ with the addition of 20 μL $Cl_2CHCOOH$ (the concentration of acid is much higher than that in the electrochemical reaction).

4. X-ray crystal structure analysis

Table S2. Crystal data and structure refinement of [**1**Cl]$^{+}$.

	[**1**Cl]$^{+}$
Empirical formula	$C_{39}H_{45}Cl_1CoF_6NiP_3S_2 \cdot CH_2Cl_2$
Formula weight	1022.79
Temperature / K	293(2)
Crystal system	monoclinic
Space group	$P2_1/n$
a / Å	14.1464(3)
b / Å	13.3562(4)
c / Å	25.4313(7)
α / °	90
β /°	103.789(2)
γ / °	90
Volume / Å^3	4666.6(2)
Z	4
ρ_{calc} / g cm^{-3}	1.456
μ / mm^{-1}	7.121
F(000)	2096.0
2Θ range for data collection / °	7.158 to 154.344
Index ranges	$-17 \le h \le 14$,<br>$-16 \le k \le 9$,<br>$-32 \le l \le 32$
Reflections collected	28729
Independent reflections	9191 [R_{int} = 0.0606, R_{sigma} = 0.0572]
Data/restraints/parameters	9191/60/510
Goodness-of-fit on F^2	1.067
Final R indexes [I>2σ (I)]	R_1 = 0.0956, wR_2 = 0.2144
Final R indexes [all data]	R_1 = 0.1301, wR_2 = 0.2377
Largest diff. peak/hole / e Å^{-3}	1.18/-1.07

Table S3. Crystal data and structure refinement of **1**.

	**1**
Empirical formula	$C_{39}H_{45}P_2S_2CoNi$
Formula weight	757.45
Temperature / K	293(2)
Crystal system	monoclinic
Space group	$P2_1/n$
a / Å	12.47350(19)
b / Å	15.4030(2)
c / Å	19.4262(3)
α / °	90
β /°	106.4521(17)
γ / °	90
Volume / $Å^3$	3579.54(10)
Z	4
ρ_{calc} / g cm^{-3}	1.406
μ / mm^{-1}	6.414
F(000)	1584.0
2Θ range for data collection / °	7.446 to 141.754
Index ranges	-15≤h≤15, -18≤k≤18, -23≤l≤22
Reflections collected	13897
Independent reflections	6782 [R_{int} = 0.0276, R_{sigma} = 0.0373]
Data/restraints/parameters	6782/0/411
Goodness-of-fit on F^2	1.038
Final R indexes [I>2σ (I)]	R_1 = 0.0370, wR_2 = 0.0881
Final R indexes [all data]	R_1 = 0.0432, wR_2 = 0.0926
Largest diff. peak/hole / e $Å^{-3}$	0.967/-0.625

Table S4. Crystal data and structure refinement of [**1**H]$^+$.

	[**1**H]$^+$
Empirical formula	$C_{39}H_{46}BCoF_4NiP_2S_2 \cdot 2CH_2Cl_2$
Formula weight	1014.11
Temperature / K	293(2)
Crystal system	triclinic
Space group	P-1
a / Å	10.9745(5)
b / Å	13.3310(6)
c / Å	17.0646(8)
α / °	78.527(4
β /°	79.830(4)
γ / °	69.506(4)
Volume / Å^3	2276.23(19)
Z	2
ρ_{calc} / g cm^{-3}	1.480
μ / mm^{-1}	7.431
F(000)	1042.0
2Θ range for data collection / °	8.262 to 153.946
Index ranges	-13≤h≤10,<br>-16≤k≤16,<br>-20≤l≤21
Reflections collected	23062
Independent reflections	9066 [R_{int} = 0.0523, R_{sigma} = 0.0590]
Data/restraints/parameters	9066/0/514
Goodness-of-fit on F^2	1.061
Final R indexes [I>2σ (I)]	R_1 = 0.0715, wR_2 = 0.1854
Final R indexes [all data]	R_1 = 0.0874, wR_2 = 0.2049
Largest diff. peak/hole / e Å^{-3}	2.59/-1.65

Table S5. Crystal data and structure refinement of [**1**]$^+$.

	[**1**]$^+$
Empirical formula	$C_{39}H_{45}P_2S_2CoNiBF_4 \cdot CH_2Cl_2$
Formula weight	929.18
Temperature / K	192.99(10)
Crystal system	monoclinic
Space group	$P2_1/n$
a / Å	12.9111(2)
b / Å	20.1158(4)
c / Å	16.5430(3)
α / °	90
β /°	99.543(2)
γ / °	90
Volume / Å^3	4237.04(13)
Z	4
ρ_{calc} / g cm^{-3}	1.457
μ / mm^{-1}	6.797
F(000)	1916.0
2Θ range for data collection / °	4.035 to 74.494
Index ranges	$-15 \leq h \leq 16$, $-25 \leq k \leq 21$, $-19 \leq l \leq 20$
Reflections collected	29630
Independent reflections	8423 [R_{int} = 0.0573, R_{sigma} = 0.0572]
Data/restraints/parameters	8423/0/483
Goodness-of-fit on F^2	1.026
Final R indexes [I>2σ (I)]	R_1 = 0.0673, wR_2 = 0.1677
Final R indexes [all data]	R_1 = 0.0850, wR_2 = 0.1838
Largest diff. peak/hole / e Å^{-3}	1.404/-1.213

Table S6. Crystal data and structure refinement of [**1**(NCMe)]$^{2+}$.

	[**1**(NCMe)]$^{2+}$
Empirical formula	$C_{41}H_{48}NP_2S_2B_2F_8CoNi·4CH_3CN$
Formula weight	1136.34
Temperature / K	172.99(10)
Crystal system	orthorhombic
Space group	$Pca2_1$
a / Å	21.9622(9)
b / Å	11.6996(4)
c / Å	20.9046(7)
α / °	90
β /°	90
γ / °	90
Volume / Å^3	5371.4(3)
Z	4
ρ_{calc} / g cm^{-3}	1.405
μ / mm^{-1}	0.863
F(000)	2352.0
2Θ range for data collection / °	6.842 to 49.996
Index ranges	-25≤h≤26,<br>-13≤k≤12,<br>-22≤l≤24
Reflections collected	27156
Independent reflections	8897 [R_{int} = 0.0466, R_{sigma} = 0.0466]
Data/restraints/parameters	8897/0/641
Goodness-of-fit on F^2	1.040
Final R indexes [I>2σ (I)]	R_1 = 0.0354, wR_2 = 0.0822
Final R indexes [all data]	R_1 = 0.0417, wR_2 = 0.0852
Largest diff. peak/hole / e Å^{-3}	0.68/-0.37

Table S7. Selected bond distances (Å) of [**1**Cl]$^+$, **1** and [**1**H]$^+$.

	[**1**Cl]$^+$	**1**	[**1**H]$^+$
Co1–Ni1	2.9257(16)	2.4722(5)	2.5557(10)
Co1–S1	2.252(2)	2.1947(6)	2.2209(13)
Co1–S2	2.259(2)	2.1820(7)	2.2252(13)
Ni1–S1	2.258(2)	2.2645(7)	2.2221(13)
Ni1–S2	2.2470(19)	2.2268(7)	2.2470(14)
Ni1–P1	2.158(2)	2.1450(7)	2.1459(14)
Ni1–P2	2.1720(19)	2.1605(7)	2.1578(13)
Co1–X	2.316(2), X = Cl	—	1.45(5), X = H
Ni1–X	2.588(2), X = Cl	—	1.91(5), X = H
Co1–Cp* plat	1.699	1.686	1.664

Table S8. Selected bond distances (Å) of [**1**]$^+$ and [**1**(NCMe)]$^{2+}$.

	[**1**]$^+$	[**1**(NCMe)]$^{2+}$
Co1−Ni1	2.6572(10)	3.005
Co1−S1	2.2099(12)	2.2465(13)
Co1−S2	2.2154(13)	2.2633(13)
Ni1−S1	2.2389(12)	2.2370(13)
Ni1−S2	2.2293(12)	2.2395(12)
Ni1−P1	2.1799(12)	2.1708(14)
Ni1−P2	2.1779(12)	2.1596(13)
Co1−X	—	1.942(4), X = N
Co1−Cp* plat	1.693	1.695

Table S9. Selected angles (deg) of the NiCo complexes.

	[**1**Cl]$^{+}$	[**1**H]$^{+}$	**1**	[**1**]$^{+}$	[**1**(NCMe)]$^{2+}$
Co1–S1-Ni1	80.88(7)	70.23(4)	67.32(2)	73.35(4)	84.19(5)
Co1–S2-Ni1	80.97(7)	69.70(4)	68.21(2)	73.43(4)	83.74(4)
S1–Co1–S2	83.54(8)	88.03(5)	93.22(2)	88.85(5)	83.18(5)
S1–Ni1–S2	83.68(7)	87.46(5)	90.17(2)	87.77(4)	83.94(5)
P1–Ni1–P2	87.27(8)	87.42(5)	89.80(3)	87.28(5)	88.14(5)
Co1–X-Ni1	73.03(7), X = Cl	98.331, X = H	—	—	76.842 X = N

References

(1) M. Schmidt, G. G. Hoffmann, *J. Organomet. Chem.,* 1977, **124***,* C5–C8.

(2) T. Yoshino, H. Ikemoto, S. Matsunaga and M. Kanai, *Angew. Chem. Int. Ed.*, 2013, **52**, 2207–2211.
